# Sex differences in a mouse model of radiation-induced cardiomyopathy

**DOI:** 10.1186/s13293-026-00998-3

**Published:** 2026-10-08

**Authors:** Emily R. Whelan, Evan E. Perona, Jessica J. Fliess, S. Christian Kocsis, Gabriel J. Weigel, Elizabeth J. McCabe, Charwan Hamilton, Damian N. Di Florio, Varsini Balamurugan, Danielle J. Beetler, Logan P. Macomb, Max W. Strandes, Frances C. Wilson, Emma F. Murphy, Ashley A. Darakjian, Molly M. Watkins, Alayna M. Puls, Cameron J. Hartmoyer, Natalie Bonvie-Hill, Sami Khatib, Chris J. McLeod, Jordan C. Ray, Michael D. Story, Steven M. Herchko, Bradford S. Hoppe, DeLisa Fairweather

**Affiliations:** 1 Department of Cardiovascular Medicine, Mayo Clinic, Jacksonville, FL USA; 2 Center for Clinical and Translational Science, Mayo Clinic, Rochester, MN USA; 3 Mayo Clinic Graduate School of Biomedical Sciences, Mayo Clinic, Jacksonville, FL USA; 4 Department of Radiation Oncology, Mayo Clinic, Jacksonville, FL USA; 5 Department of Immunology, Mayo Clinic, Jacksonville, FL USA

**Keywords:** Radiation-induced heart disease, Sex differences, Vascular damage, Mitochondrial dysfunction, Inflammation, Fibrosis, Dilated cardiomyopathy

## Abstract

**Background:**

Radiation-induced heart disease (RIHD) may develop months to years following radiation therapy for cancers near the heart such as lung or breast cancer. Several mouse models of RIHD exist to study the pathogenesis of disease, but to our knowledge no studies have examined whether sex differences exist in RIHD in mice.

**Methods:**

Female and male BALB/cJ mice received a single whole-heart dose of 22 Gy (Gy) radiation via a C5 collimator vs. sham controls that received 0 Gy, anesthesia and were placed in the X-ray machine for the same length of time. Endpoints were examined at 24 h (innate), day 10 (acute) and day 35 (chronic) post irradiation.

**Results:**

Females and males developed dsDNA breaks from radiation at day 10 but females had more damage than males (*p* < 0.001). Vessel area and wall thickness were increased by radiation at day 10 and 35 but vessel area was greater in irradiated males than females at day 35 (*p* = 0.003). Bulk RNA sequencing of the heart at day 10 revealed that females and males developed similar gene pathway responses to radiation by upregulating cellular repair pathways and developing mitochondrial dysfunction. However, females upregulated the master regulator of mitochondrial function, Pgc1α (*p* = 0.0001), suggesting possible improved mitochondrial repair in females. By day 35, females developed mild cardiomyopathy while males developed myocardial and perivascular fibrosis and inflammatory dilated cardiomyopathy (iDCM).

**Conclusions:**

We observed sex differences in most cardiac parameters that we examined following radiation exposure. BALB/c males exposed to 22 Gy radiation developed iDCM by day 35 while females developed mild cardiomyopathy. Further research is needed to better understand mechanisms driving sex differences in RIHD using translational animal models.

**Supplementary Information:**

The online version contains supplementary material available at https://doi.org/10.1186/s13293-026-00998-3.

## Abstract (plain language summary)

Radiation therapy used to treat cancers near the heart, such as breast and lung cancer, can sometimes cause heart damage months or years later, a condition known as radiation-induced heart disease (RIHD). Although mouse models are commonly used to study RIHD, little is known about whether males and females respond differently to radiation exposure. In this study, male and female BALB/cJ mice received a single radiation dose directed at the heart and were compared with non-irradiated control mice. Researchers examined early and later effects of radiation over a 35-day period. Both sexes experienced radiation-induced DNA damage in heart cells, but females showed greater DNA damage than males. Radiation also increased blood vessel size and heart enlargement in both sexes, although these changes were more pronounced in males. Gene expression analysis showed that males and females activated many of the same repair pathways after radiation and both showed reduced mitochondrial function. However, females increased expression of Pgc1α, a key regulator of mitochondrial health, suggesting they may have a greater ability to repair mitochondrial damage. By 35 days after radiation exposure, males developed heart fibrosis and inflammatory dilated cardiomyopathy, while females developed mild heart disease without evidence of inflammation or fibrosis. These findings demonstrate important sex-specific differences in the cardiac response to radiation and highlight the need for further research to understand the biological mechanisms underlying these differences.

**Supplementary Information:**

The online version contains supplementary material available at https://doi.org/10.1186/s13293-026-00998-3.

## Highlights

First translational study demonstrating sex differences in RIHD in mice.

Cardiac DNA damage, mitochondrial dysfunction, vacuole formation, and increased vessel size occurred in both sexes after radiation exposure.

Males developed worse cardiomyopathy than females with elevated IL-1β, cardiac remodeling and inflammatory DCM.

Females upregulated Pgc1α which may have improved their heart function.

**Supplementary Information:**

The online version contains supplementary material available at https://doi.org/10.1186/s13293-026-00998-3.

## Introduction

Radiation therapy is a common treatment for patients with thoracic malignancies such as breast cancer, lung cancer, and mediastinal lymphoma. Although highly conformal techniques, such as intensity modulated radiation therapy and proton therapy, are used to minimize collateral damage to the heart when irradiating thoracic cancers, patients receiving radiation therapy are at risk of developing radiation-induced heart disease (RIHD) months to years later [1, 2]. RIHD can present clinically as cardiomyopathy/ dilated cardiomyopathy (DCM), acute coronary syndrome with vascular damage, congestive heart failure, pericarditis, myocardial fibrosis, conduction disorders, and/or valvular disease [3–5]. RIHD is most often a diagnosis of exclusion, and for this reason is likely to be underdiagnosed [6]. Thus, there is a need to identify early diagnostic biomarkers that predict the risk of cardiomyopathy following radiation therapy for earlier diagnosis.

Men have a higher overall prevalence of cardiovascular diseases (CVDs) than women in the US (52.4% vs. 44.8%) [7]. Many CVDs exhibit sex differences in prevalence, with conditions such as hypertension, atherosclerosis, myocarditis, and DCM occurring more often in men, whereas relatively few conditions, including cardiovascular-kidney-metabolic (CKM) syndrome, occur more often in women [8–10]. Sex differences also exist in heart failure (HF), with males primarily developing HF with reduced ejection fraction (HFrEF) while females are more likely to develop HF with preserved ejection fraction (HFpEF) [9, 11, 12]. Khalid et al. conducted a meta-analysis of 10 studies of 13,975 patients following radiation therapy, 41% females and 59% males, and found a higher RIHD prevalence and mortality in females compared to males [13]. Interestingly, they found that mortality from RIHD was higher in elderly women in their analysis [13]. Sex hormones and chromosomes promote sex differences in cardiac physiology and the immune response that drives cardiopathology [8, 9, 14, 15]. Further research is needed to better understand whether sex differences exist in RIHD.

Numerous animal models have examined radiation-induced cardiotoxicity using C57BL/6 males [16–19] or females [20–23]. However, few models have compared sex differences in RIHD. Andruska et al. examined whether sex differences exist in male vs. female inbred Dahl salt sensitive rats using 24 Gray (Gy) in a single fraction to the whole heart using a 1.5 cm collimator [24]. They found that 100% of females developed pericardial effusion and heart failure while only 14% of males developed this form of cardiomyopathy [24]. They identified that collateral damage to the lungs was greater in females because of their smaller heart size and when they increased the size of the collimator to 2.0 cm in males there was no sex difference in pericardial effusion or mortality by sex. Because rats have few resources like knockout animals or antibodies available to study mechanisms of disease, a mouse model to study sex difference in RIHD is needed. In this study we exposed BALB/c mice to 22 Gy using a sex-specific dosimetry plan to the whole heart which resulted in very low lung collateral damage in order to study sex differences in RIHD.

## Results

### Cardiomyopathy model

**Fig. 1:**
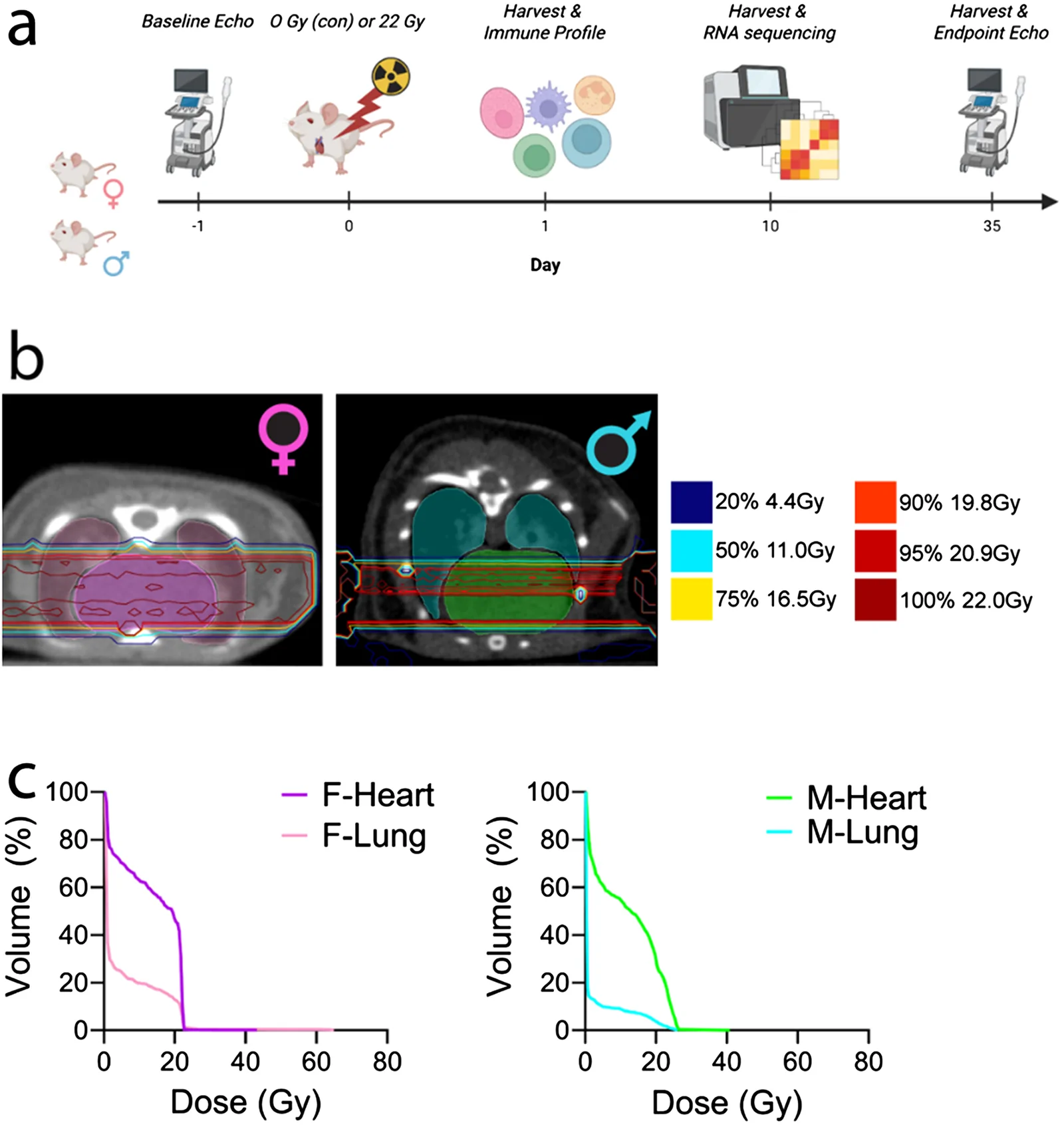
Study plan and dosimetric protocol. **(a)** Experimental timeline for male and female BALB/c mice. **(b)** A representative plan for whole heart radiation using 22 Gy for female (*left*) and male (*right*) BALB/c mice showing the axial section. Females and males were analyzed in separate experiments using sex-specific dosimetric plans to account for differences in heart size between sexes. The heart (purple for females, green for males) was irradiated using a parallel opposed method in the axillary region (beams are 180 degrees apart and parallel to each other so that the beam converges to the isocenter). Mice were placed in a feet-first prone position in relation to the gantry which was equipped with a 5-millimeter circular collimator (C5). The isocenter of radiation is aimed medial to the left and right ventricles and superior to the apex of the heart. **(c)** Representative image of the dose-volume histogram (DVH) showing the percentage of heart (purple for females, green for males) and lung (pink for females, blue for males) receiving 22 Gy radiation

BALB/c mice were chosen for this study because C57BL/6 mice are resistant to developing cardiomyopathy and do not display sex differences in several mouse models of cardiomyopathy [8, 9, 25–28]. Female and male BALB/c mice were randomly assigned to receive 0 Gy (control) or 22 Gy based on preliminary data indicating that this was an effective dose for developing cardiomyopathy at day 35 after irradiation (data not shown). Because male hearts are larger than female hearts, we ensured that the same dose of radiation was administered to the heart for each sex by creating a sex-specific dosimetric plan (Supplemental Table 1). The plan prescribed 22 Gy to the midplane of two parallel opposed lateral beams with the heart at the center, which helped reduce exposure to the lungs (Fig. 1). Based on previous experience with murine models of cardiomyopathy [8, 9, 29, 30], three time points were selected as endpoints: 24 hours (h) (innate), day 10 (acute) and day 35 (chronic) (Fig. 1a). At day 10 we confirmed the presence of radiation-induced double-stranded DNA (dsDNA) damage by measuring γH2A.x. Mice received a baseline echocardiogram (echo) on day − 1 and an endpoint echo at day 35 to detect cardiomyopathy (Fig. 1a). Quantitative RT-PCR (qPCR) was performed at 24 h, day 10 and 35 after radiation.

#### Sex-specific treatment plan

Figure 1b shows a representative treatment plan image of a female and male whole heart radiation aimed medial to the left and right ventricles and superior to the apex of the heart. A representative dose-volume histogram (DVH) showing the percentage of the heart and lung receiving 22 Gy of radiation is presented in Fig. 1c. For females, the heart and lung are shown in purple and pink, respectively; for males, the heart and lung are shown in green and blue, respectively. The DVH indicates very low collateral radiation exposure to the lungs for both sexes.

### More DNA double strand breaks at 10 days in females

**Fig. 2:**
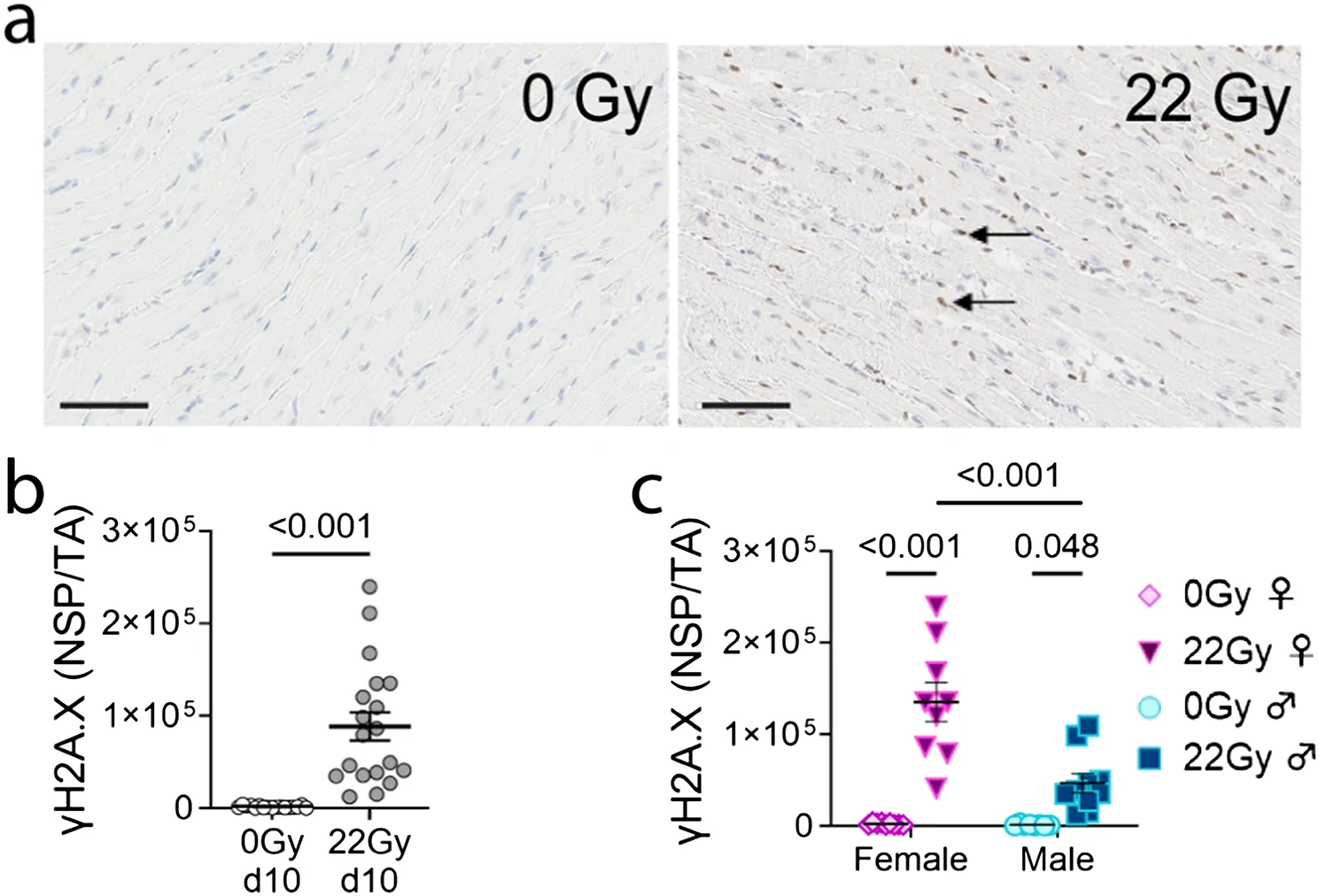
Sex differences in dsDNA breaks at day 10 post radiation. Male and female BALB/c mice were irradiated with sex-specific dosimetric plans and IHC performed on hearts to measure dsDNA breaks using antibodies against γH2A.x. **(a)** Representative images of γH2A.x IHC staining of 0 and 22 Gy (bars = 200 μm). Positive staining is brown on a blue background (arrows). dsDNA breaks were quantified at day 10 from γH2A.x IHC and shown as the number of strong positives (NSP) normalized to the total area (TA) for female and male mice **(b)** combined or **(c)** separated by sex that received 0 Gy (0Gy) vs. 22 Gy (22Gy) (*n* = 8–10/group). **b)** Data shown as the mean ± SEM for *n* = 8–10 mice/group using an unpaired Mann-Whitney rank test. **c)** 2-way ANOVA, radiation *p* < 0.001, sex *p* < 0.001, interaction *p* = 0.001, p values obtained from Sidak’s multiple comparisons test

To examine whether dsDNA breaks were present at day 10 after irradiation we examined γH2A.x staining using immunohistochemistry (IHC). Representative images of control 0 Gy vs. 22 Gy are shown in Fig. 2a. Combining females and males, we observed a significant increase in dsDNA breaks in the heart of mice treated with 22 Gy compared to 0 Gy controls (*p* < 0.001) (Fig. 2b) at day 10. Compared to controls, we found greater dsDNA breaks in females treated with 22 Gy vs. female controls (*p* < 0.001) and in males treated with 22 Gy vs. male controls (*p* = 0.048) (Fig. 2c). By sex, cardiac DNA damage indicated by elevated γH2A.x was greater in females than males (*p* < 0.001) (Fig. 2c). 2-way ANOVA revealed significant differences for radiation *p* < 0.001, sex *p* < 0.001, and an interaction of radiation and sex *p* = 0.001. Thus, more DNA double strand breaks were present at 10 days in the heart of females versus males exposed to radiation.

### Radiation induced vascular damage in both sexes, with greater vessel area in males at day 35

**Fig. 3:**
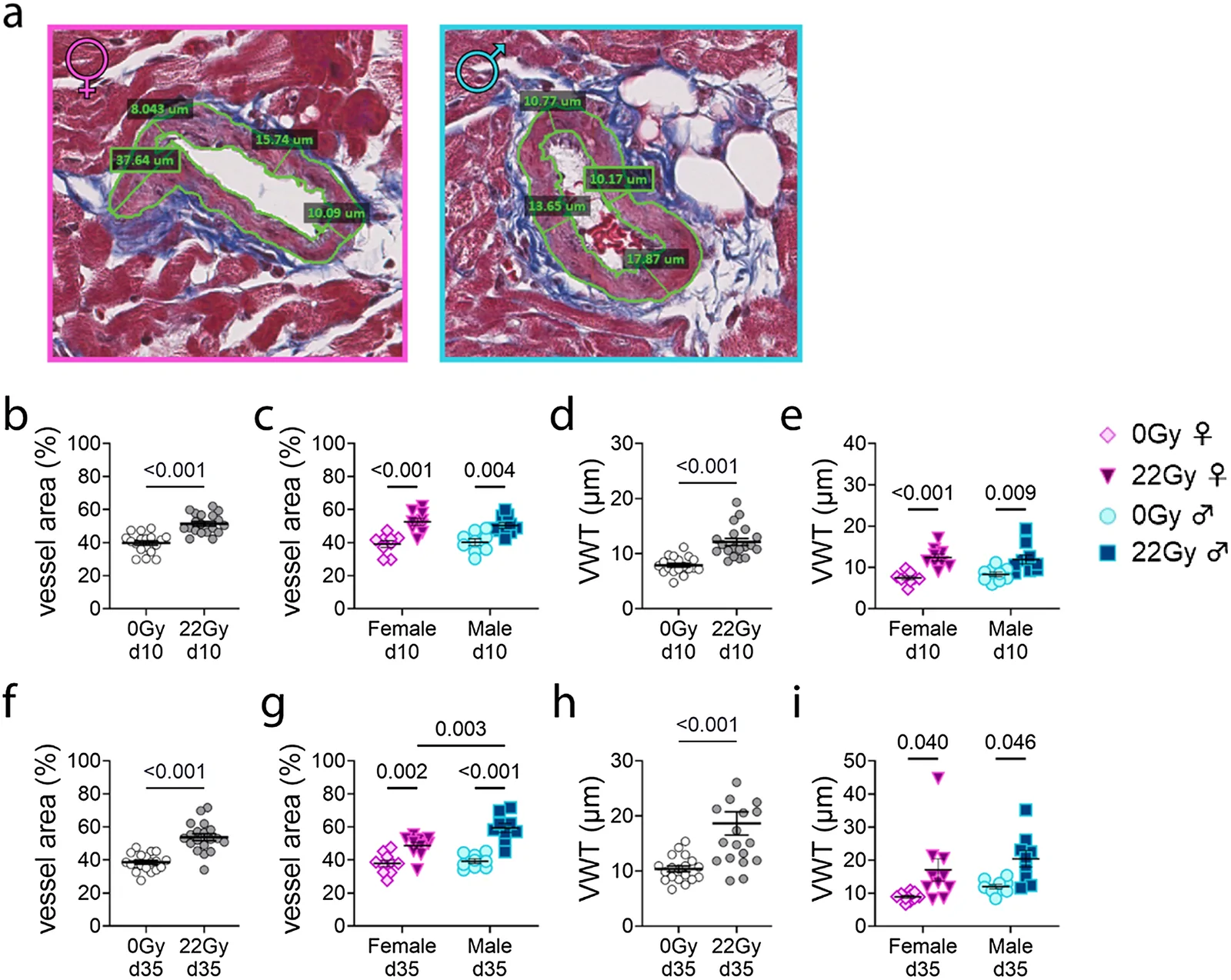
Radiation induces vascular damage at day 10 and 35 in females and males. Male and female BALB/c mice were irradiated with sex-specific dosimetric plans and vessel size determined from Masson’s trichrome stained slides. **a)** Representative photos of vessels for females (pink) and males (blue) are shown using Masson’s trichrome stain. Green lines indicate inner and outer vessel tracings used to determine vessel area while 4 lines were used/vessel to determine vessel wall thickness (VWT). Female and male BALB/c mice received 0 Gy (0Gy) or 22 Gy (22Gy) at day 0 and were examined at **b-e)** day 10 (d10) and **f-i)** day 35 (d35) after irradiation for vascular damage. Vessel area and VWT are shown for males and females combined (b, d, f, h) or separated by sex (c, e, g, i). Data show the mean ± SEM for 8–10 mice/group. **b**,** d**, **f**,** h)** P values obtained using unpaired Mann-Whitney rank tests. **c**,** e**, **g**,** i)** A two-way ANOVA was used to assess the interaction between radiation and sex, with *p* values obtained using Sidak’s multiple-comparisons test

To determine whether cardiac vessels were damaged by 22 Gy irradiation on day 10 and 35, we measured vessel area (%) and vessel wall thickness (VWT). Representative photos of vessels for females (*left*) and males (*right*) (Fig. 3a) are shown using Masson’s trichrome histology stain. Green lines indicate inner and outer vessel tracings used to determine vessel area while 4 lines transecting each vessel were used to determine vessel wall thickness.

#### Vessel wall area and thickness at day 10 and 35

At day 10 post radiation, we found increased vessel area in mice treated with 22 Gy vs. controls for females and males combined (*p* < 0.001) and for females and males individually (*p* < 0.001, *p =* 0.004; respectively) vs. same sex controls (Fig. 3b, c) (2-way ANOVA, radiation *p* < 0.001, sex *p* = 0.78, interaction *p* = 0.40). Similarly, we found that 22 Gy increased VWT at day 10 compared to controls for females and males combined (*p* < 0.001) (Fig. 3d), and for females (*p* < 0.001) and males (*p* = 0.009) compared to same sex controls (Fig. 3e) (2-way ANOVA, radiation *p* < 0.001, sex *p* = 0.81, interaction *p* = 0.40).

Vessel damage persisted to day 35 after radiation where we found increased vessel area in mice treated with 22 Gy vs. controls for females and males combined (*p* < 0.001) and for females (*p* = 0.002) and males (*p* < 0.001) individually vs. same sex controls (Fig. 3f, g). However, at day 35 post radiation males had larger vessel areas than females (*p* = 0.003) (Fig. 3g) (2-way ANOVA, radiation *p* < 0.001, sex *p* = 0.006, interaction *p* = 0.030).

Similarly, we found that 22 Gy increased VWT at day 35 compared to controls for females and males combined (*p* < 0.001) (Fig. 3h). Similar to day 10, we found that VWT was increased in 22 Gy vs. controls for females (*p* = 0.040) and males (*p* = 0.046) individually vs. same sex controls (Fig. 3i) (2-way ANOVA, radiation *p* < 0.001, sex *p* = 0.15, interaction *p* = 0.95). Thus, overall radiation induced vessel damage in both sexes, but males had a greater vessel area than females at day 35 after radiation.

### Irradiated male hearts were larger than females but weighed the same as female and male controls at day 35

To further determine whether sex differences existed in cardiomyopathy after 22 Gy exposure, we examined heart weight (HW) normalized to tibia length (TL) ratio.

#### Body and heart weights at day 10

**Fig. 4:**
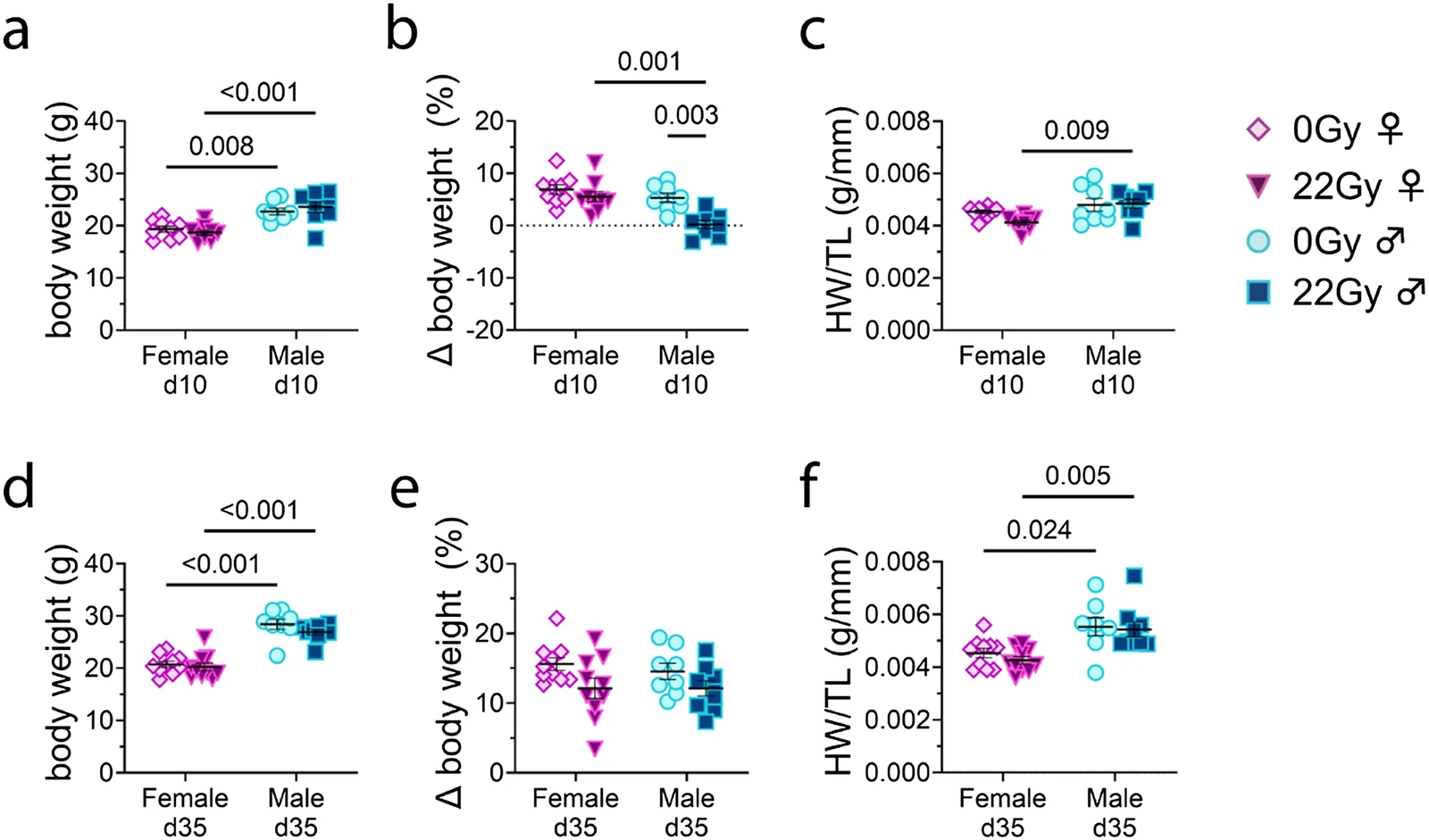
Irradiated males gain less body weight by day 10, with similar weight gain to controls by day 35 and no change in heart weight vs. controls at day 35. Mice received 0 Gy (0Gy) vs. 22 Gy (22Gy) irradiation at day 0 and were assessed at **a-c)** day 10 (d10) and **d-f)** day 35 (d35) for body weight, change in body weight, and heart weight (HW) to tibia length (TL). Data shown as mean ± SEM for 8–10 mice/group. A two-way ANOVA was used to assess the interaction between radiation and sex, with *p* values obtained using Sidak’s multiple-comparisons test

At day 10, control and irradiated males weighed more than females (0 Gy *p* = 0.008, 22 Gy *p* < 0.001) (Fig. 4a) (2-way ANOVA, radiation *p* = 0.044, sex *p* < 0.001, interaction *p* = 0.28). At day 10 males that received 22 Gy gained less body weight than male 0 Gy controls (*p* = 0.003) or 22 Gy females (*p* = 0.001) (Fig. 4b) (2-way ANOVA, radiation *p* = 0.001, sex *p* < 0.001, interaction *p* = 0.053). At day 10, males exposed to 22 Gy had a significantly greater HW/TL ratio compared to females (*p* = 0.009) (Fig. 4c), indicating that males had larger hearts at day 10 following radiation exposure than females (2-way ANOVA, radiation *p* = 0.25, sex *p* = 0.003, interaction *p* = 0.14).

#### Body and heart weights at day 35

At day 35, males continued to weigh more than females (0 Gy *p* < 0.001, 22 Gy *p* < 0.001) (Fig. 4d) (2-way ANOVA, radiation *p* = 0.18, sex *p* < 0.001, interaction *p* = 0.48). In contrast to day 10, by day 35 body weight gain in males exposed to 22 Gy was similar to male controls (Fig. 4e). Like day 10, at day 35 males exposed to 22 Gy continued to have a significantly greater HW/TL ratio compared to females (*p* = 0.005) (Fig. 4f) (2-way ANOVA, radiation *p* = 0.45, sex *p* < 0.001, interaction *p* = 0.73). Thus, irradiated males gained less body weight by day 10, whereas by day 35 their body weight gain was similar to male controls (Fig. 4b, e). These findings primarily reflected sex differences in body weight rather than sustained radiation-associated weight loss.

### Males develop dilated cardiomyopathy by day 35

**Fig. 5:**
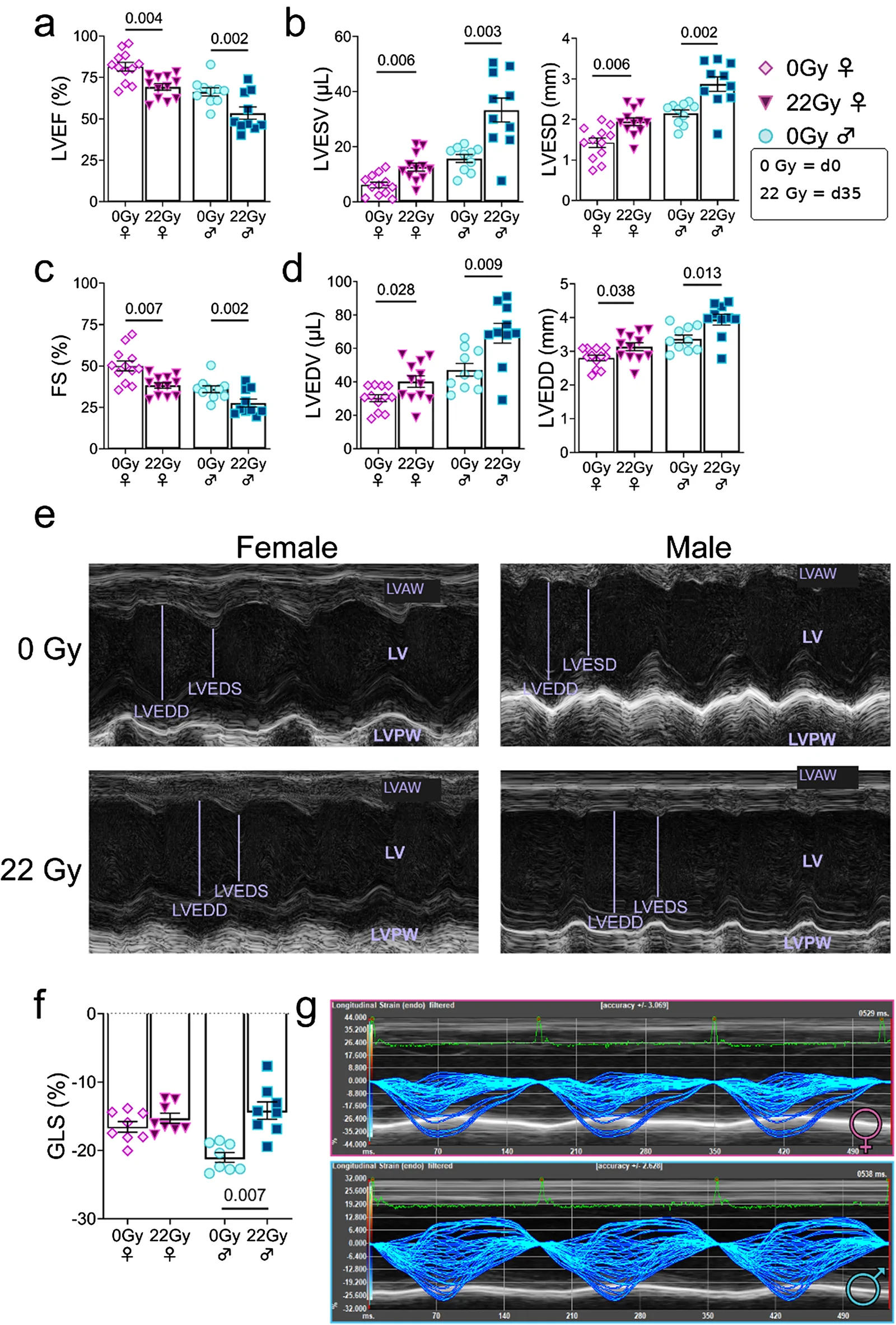
Males develop DCM by day 35. The cardiac function of female and male BALB/c mice was assessed at day 0 (baseline, 0Gy/d0) and day 35 after receiving 22 Gy (22Gy/d35) for the following parameters in separate experiments for females and males: **(a)** left ventricular (LV) ejection fraction (LVEF), **(b)** LV end systolic volume (LVESV) and diameter (LVESD), **(c)** fractional shortening (FS), **(d)** LV end diastolic volume (LVEDV) and diameter (LVEDD), **(e)** representative M-mode images of myocardium illustrating regions of the heart and where measurements were obtained showing 0 and 22 Gy females and males. **(f)** Average global longitudinal strain (GLS) measurements (*left*) and **(g)** representative images of GLS desynchrony using speckle tracking (blue lines) of 48 locations of the endocardial wall inside of the LV over three cardiac cycles in females (*top*) and males (*bottom*) are shown at day 35. **a-d,f)** Data show the mean ± SEM for *n* = 8–13 mice/group, p values obtained using Wilcoxon matched-pairs signed rank test. Abbreviations: FS, fractional shortening; GLS, global longitudinal strain; LVAW, left ventricular anterior wall; LV, left ventricular; LVEDD, LV end-diastolic diameter; LVEDV, LV end-diastolic volume; LVEF, LV ejection fraction; LVESD, LV end-systolic diameter; LVESV, LV end-systolic volume; LVPW, left ventricular posterior wall

Because of the high variability in heart size and cardiac function observed in normal female and male BALB/c mice [31], we performed a baseline echo at day − 1 before irradiation. After irradiation, we performed paired analyses comparing baseline 0 Gy to 22 Gy for each mouse using Wilcoxon matched-pairs rank test by sex (not between sexes) but graphed sexes together. Heart rate was monitored during echocardiography; the group distributions are shown in Supplementary Fig. 1a. At baseline (day − 1), female and male mice displayed normal ventricular function using echo (Fig. 5).

#### Cardiac function at day 35

After 22 Gy irradiation, both females and males had a significant reduction in left ventricular (LV) ejection fraction (EF) (LVEF) at day 35 compared to baseline (*p* = 0.004, *p* = 0.002; respectively) (Fig. 5a). However, the irradiated male group had a mean LVEF of approximately 50%, with several individual males at or below 50%, whereas the female group mean remained above 50% **(**Fig. 5a).

#### Dilation at day 35

Females and males also developed significant increases in LV end systolic volume (LVESV) (*p* = 0.006, *p* = 0.003; respectively) and diameter (LVESD) (*p* = 0.006, *p* = 0.002; respectively) following irradiation (Fig. 5b), indicating dilatation. Fractional shortening (FS) was also decreased in females and males after irradiation (*p* = 0.007, *p* = 0.002; respectively) (Fig. 5c), indicating a reduced ability to pump blood (i.e., contract). Females and males had a significant increase in LV end diastolic volume (LVEDV) (*p* = 0.028, *p* = 0.009; respectively) and diameter (LVEDD) (*p* = 0.038, *p* = 0.013; respectively) (Fig. 5d), but the larger values in Fig. 5b and d in males were indicative of DCM while the values in females did not indicate DCM. Unfortunately, because females and males were not examined within the same experiment, direct comparisons were not conducted. High LVESD and LVEDD measurements during echocardiography are accepted indicators of DCM [32, 33]. Representative M-mode images demonstrate how the measurements were obtained and depict greater ventricular dilation in males than females after 22 Gy exposure (Fig. 5e). Supplementary Fig. 1 provides additional echocardiography measurements where we found no differences between groups by radiation or sex.

#### Baseline vs. day 35 0 Gy controls

Because we compared irradiated mice to baseline (day 0) rather than day 35 controls, we examined whether differences existed in cardiac functional echo parameters comparing 0 Gy controls at baseline (day 0) to 0 Gy controls at endpoint (day 35) to determine whether age (i.e., 35 days) affected cardiac parameters. We found that there were no significant differences for any parameter comparing these two controls except for left ventricular posterior wall thickness at systole (LVPW;s) in males (baseline 0.94 ± 0.09 vs. 1.28 ± 0.07, *p* = 0.015) (Supplemental Table 2). These data suggest that using baseline mice (day 0) instead of aged control mice (day 35) did not, in general, significantly affect our findings.

#### Global longitudinal strain

Global longitudinal strain (GLS) is a sensitive indicator of cardiac stress (i.e., difficulty pumping) in mice and humans, with a percentage of -17 to -20% considered to be normal [31, 34, 35]. Thus, after irradiation only males had significantly worse GLS (*p* = 0.007) (closer to 0) compared to baseline (Fig. 5f). Radiation exposure also led to a loss of synchronous cardiac contractions, which can be visualized in the strain curves in Fig. 5g, with females having more synchrony (blue strands closer together) (*top*) than males (separate blue strands) (*bottom*) at 22 Gy. Thus, male BALB/c mice given 22 Gy developed more cardiac strain, reduced ejection fraction and larger dilatation measures indicative of DCM by day 35 after irradiation while females developed mild cardiomyopathy.

### Radiation leads to cardiac inflammation in males by day 35

To determine whether radiation damage to the heart led to cardiac inflammation, we assessed whether there was a cardiac inflammatory cell infiltration using histology and qPCR in male and female mice that received 0 or 22 Gy.

#### Changes in immune infiltrate over time

**Fig. 6:**
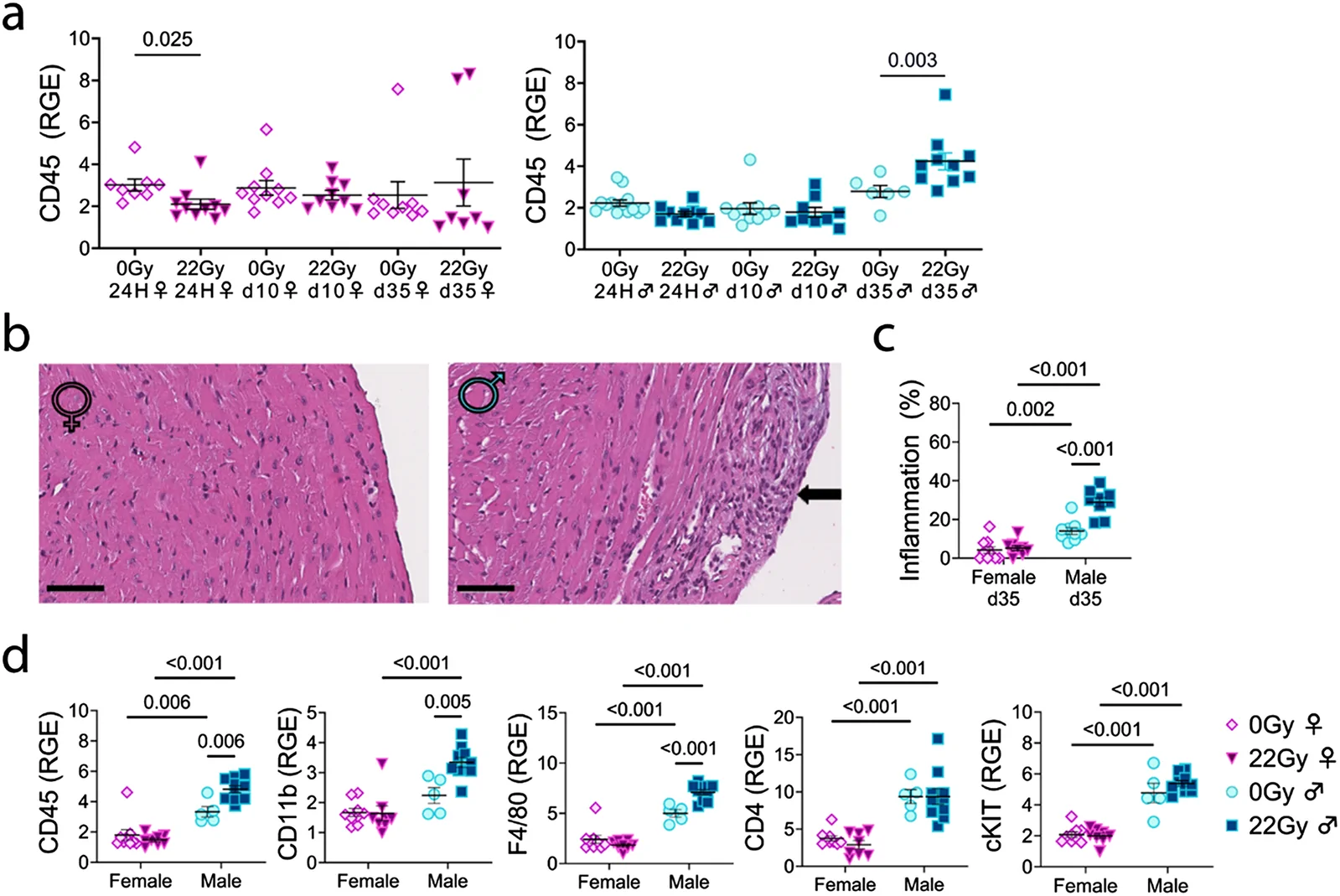
Radiation induces cardiac inflammation at day 35 in males. **(a)** In separate experiments female (pink) and male (blue) BALB/c mice received 0 (0Gy) or 22 (22Gy) Gy on day 0 and hearts examined at 24 h (24H), day 10 (d10) or day 35 (d35) after irradiation by qPCR for relative gene expression (RGE) of CD45, a common leukocyte marker, normalized to the housekeeping gene *Hprt*. **(b)** Representative H&E histology are shown for a female and male heart at day 35 after irradiation (bar = 60 μm). Arrow indicates inflammation. **(c)** Cardiac inflammation at day 35 after irradiation was quantified using a microscope eye-piece grid normalized to the size of the heart. **(d)** Hearts at day 35 after irradiation were analyzed by qPCR for mRNA expression of immune-cell-associated markers: CD45 (leukocytes), CD11b (myeloid cells and some mast cells), F4/80 (macrophage-associated), CD4 (T helper-cell-associated), and cKit (mast-cell-associated). **a**,** c**, **d)** Data shown as mean ± SEM for 5–10 mice/group. **c**,** d)** 2-way ANOVA, p values obtained using Sidak’s multiple comparisons test

Using qPCR, we examined CD45 levels in the heart at 24 h, day 10 and 35 in females and males (timepoints analyzed on the same RT plate) (Fig. 6a). Female mice had a significant decrease in cardiac CD45 expression at 24 h post radiation (*p* = 0.025) (Fig. 6a, *left*). In contrast, males had no change in cardiac CD45 expression until day 35 when levels significantly increased (*p* = 0.003) (Fig. 6a, *right*). No increase in CD45 mRNA expression was observed at day 35 in females.

#### Immune response to the heart at day 35

Representative photos of H&E staining depicting inflammatory cells are shown at day 35 after irradiation in Fig. 6b. Cardiac infiltration was not observed in heart sections of female mice but were observed in males (clumps of purple nuclei, *arrow*) (Fig. 6b). Cardiac inflammation was further quantified using a microscope grid and normalized to the size of the heart. Histological assessment showed only low levels of inflammatory-cell infiltration in females that did not increase with radiation exposure (*p* = 0.91) while, in contrast, irradiated males had small foci of inflammation, diffuse myocardial inflammation and pericarditis compared to control males (*p* < 0.0001) (2-way ANOVA, radiation *p* < 0.0001, sex *p* < 0.0001, interaction *p* = 0.0004) (Fig. 6c).

#### Sex differences in specific cardiac immune cell types at day 35

Next, we characterized the cardiac immune infiltrate at day 35 post radiation in females and males using qPCR (sex analyzed on the same RT plate) (Fig. 6d). CD45 relative gene expression (RGE) suggested that CD45+ leu­kocytes may have been present at higher levels at 0 Gy in males than females (*p* = 0.006). However, CD45, a common leukocyte marker, showed significantly increased mRNA expression in males at 22 Gy vs. male controls (*p* = 0.006). Thus, cardiac CD45 mRNA expression was higher in males vs. females at 22 Gy (*p* < 0.001) (2-way ANOVA, radiation *p* = 0.048, sex *p* < 0.001, interaction *p* = 0.004). qPCR of CD11b, which is expressed by neutrophils, macrophages, some dendritic cells and mast cells, revealed increased CD11b mRNA expression in males with radiation vs. controls (*p* = 0.005). CD11b mRNA expression was also significantly higher in irradiated males vs. females at day 35 (*p* < 0.001) (2-way ANOVA, radiation *p* = 0.048, sex *p* < 0.001, interaction *p* = 0.004). F4/80 mRNA, a macrophage-associated marker, was significantly higher in male vs. female controls at day 35 (*p* < 0.001), increased in 22 Gy males vs. male controls (*p* < 0.001), and was higher in irradiated males vs. females at day 35 (*p* < 0.001) (2-way ANOVA, radiation *p* = 0.028, sex *p* < 0.001, interaction *p* = 0.001). CD4 mRNA expression was higher in control males than females at day 35 (*p* < 0.001) and was also higher in irradiated males than females at day 35 (*p* < 0.001) (2-way ANOVA, radiation *p* = 0.62, sex *p* < 0.001, interaction *p* = 0.61). cKit mRNA expression, a mast-cell-associated marker [36–38], was higher in control males than females at day 35 (*p* < 0.001) and was also higher in irradiated males than females at day 35 (*p* < 0.001) (2-way ANOVA, radiation *p* = 0.48, sex *p* < 0.002, interaction *p* = 0.028). Additionally, mRNA expression of CD19 (B-cell-associated), CD3 (T-cell-associated), and CD8 (cytotoxic T-cell-associated) markers was also higher in males at day 35 after radiation (data not shown). Because qPCR of individual markers does not directly quantify immune-cell numbers, these gene-expression data were interpreted together with the histologic evidence of inflammatory-cell infiltration. Combined with the echocardiography findings, the data indicate that males developed inflammatory DCM (iDCM) following irradiation.

### Radiation induces myocardial and perivascular fibrosis at day 35 in males

#### Myocardial/pericardial fibrosis at day 35

**Fig. 7:**
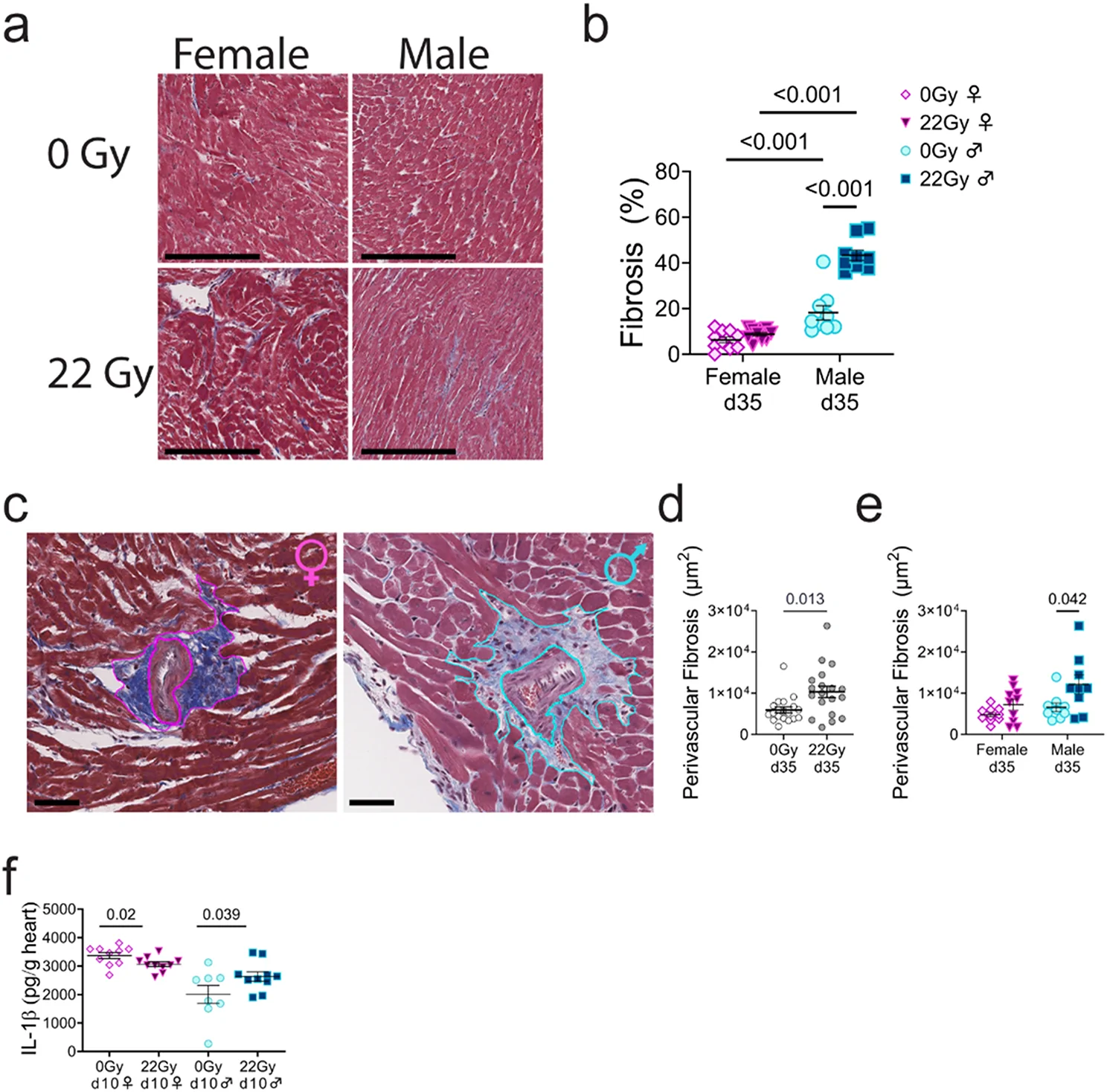
Radiation induces remodeling and fibrosis at day 35 in males and cardiac cytokine changes at day 10. Female and male BALB/c mice received 0 Gy (0Gy) or 22 Gy (22Gy) at day 0 and were examined at day 35 (d35) after irradiation for fibrosis and at day 10 (d10) for cardiac cytokine levels by ELISA. **(a)** Representative images of collagen deposition in the myocardium using Masson’s trichrome stain (Bar = 70 μm). **(b)** Quantification of fibrosis from Masson’s trichrome stained hearts using a microscope eye-piece grid normalized to the size of the heart. **(c)** Representative images of perivascular fibrosis from Masson’s trichrome slides (Bar = 50 μm). Pink (female, left) and blue (male, right) illustrate how the perivascular area of fibrosis was calculated. Quantification of perivascular fibrosis for **(d)** males and females together and **(e)** individually are shown. **(f)** ELISA of IL-1β levels in the heart at day 10 after radiation normalized to heart weight. Data shows the mean ± SEM for 8–10 mice/group. **d)** P values obtained using unpaired Mann-Whitney rank tests. **b**,** e**, **f)** 2-way ANOVA, p values obtained using Sidak’s multiple comparisons test

To determine whether radiation exposure led to fibrosis on day 10 or 35 after 22 Gy exposure, hearts were stained with Masson’s trichrome to detect collagen deposition. We did not find an increase in collagen deposition at day 10 in the heart of females or males compared to controls (data not shown). Histologically we observed increased collagen deposition along the pericardium (not shown), within the myocardium (Fig. 7a, b) and surrounding vessels (Fig. 7c, d) at day 35 after irradiation in males. Representative images of Masson’s trichrome staining for collagen deposition (blue staining indicates collagen) in the myocardium are shown in Fig. 7a. Quantification of Masson’s trichrome staining was conducted using a microscope eyepiece grid and values were normalized to the size of the heart tissue. Collagen is typically found in the heart around vessels (low level), valves, and where the myocardium joins the aortic root. We excluded valves and the aortic root area in our assessment. Quantification of cardiac fibrosis which included myocardial, perivascular and pericardial fibrosis is shown in Fig. 7b. We found that females had low background levels of fibrosis in the myocardium that did not differ between irradiated females and controls (*p* = 0.81) (Fig. 7b). Males had higher background fibrosis levels in 0 Gy controls compared to 0 Gy females (*p* = 0.001). In contrast, radiation exposure increased fibrosis in males compared to 0 Gy control males (*p* < 0.0001) (Fig. 7b). Thus, 22 Gy irradiation increased overall fibrosis in the heart of males but not females at day 35 (2-way ANOVA, radiation *p* < 0.0001, sex *p* < 0.0001, interaction *p* = 0.0001).

#### Perivascular fibrosis at day 35

Next, we specifically examined whether radiation exposure increased perivascular fibrosis using Masson’s trichrome stained slides. Representative photos are shown in Fig. 7c that depict how the perivascular fibrosis area was calculated. We found that radiation increased perivascular fibrosis in the heart at day 35 post irradiation when males and females were combined (*p* = 0.013) (Fig. 7d, *left*). But when examining the data by sex, we found that only 22 Gy males had increased perivascular fibrosis compared to male controls (*p* = 0.042) (Fig. 7c, *right*).

### Profibrotic cytokines in the heart at day 10

#### IL-1β

The cytokine interleukin (IL)-1β is an acute (day 10) driver of chronic cardiac fibrosis (day 35) after tissue damage that displays sex differences [30]. For this reason, we examined whether sex differences existed in IL-1β levels in the heart by ELISA of homogenized heart supernatant at day 10 following radiation. We found that females had reduced IL-1β levels in the heart at day 10 after radiation (*p* = 0.020), while males had increased levels (*p* = 0.039) (Fig. 7e) (unpaired t-test). IL-1β is produced from both cardiomyocytes and immune cells (i.e., macrophages, mast cells). Thus, the presence of more macrophages and mast cells in males following radiation may increase cardiac IL-1β levels leading to greater fibrosis in males at day 35.

#### Time-course of TLR4 by qPCR

Because Toll-like receptor (TLR)4 is critical for production of IL-1β, we examined TLR4 levels in the heart at 24 h, day 10 and 35 by qPCR. We found that TLR4 levels were decreased at 24 h in the heart of irradiated males compared to male controls (*p* = 0.032) (Supplemental Fig. 2a). There were no differences in TLR4 levels between controls and irradiated mice or by sex at day 10 in the heart (Supplemental Fig. 2b). But at day 35 we found that TLR4 levels were increased in the heart of males compared to females irrespective of control or radiation status (*p* < 0.001, *p* = 0.001, respectively) (Supplemental Fig. 2c). This resembles the immune cell pattern observed in the heart for CD45 by qPCR in Fig. 6, suggesting that the fluctuation in TLR4 expression may in part parallel changes in immune-cell-associated gene expression in the heart.

#### TNFα and TGFβ1 at day 10

We examined two other cytokines known to drive remodeling and fibrosis in the heart, tumor necrosis factor-α (TNFα) and transforming growth factor (TGF)β1, by ELISA to see if they displayed sex difference in the heart at day 10 post irradiation. We found that TNFα was significantly decreased at day 10 in females after irradiation (*p* = 0.020) but there was no change in males (Supplemental Fig. 3a). We found no significant difference in TGFβ1 levels in the heart of either sex at day 10 after irradiation (Supplemental Fig. 3b).

### RNA sequencing of the heart reveals radiation-induced mitochondrial dysfunction in females and males at day 10 and little gene activity at day 35

#### RNAseq at day 35

To better understand mechanisms that drive chronic cardiomyopathy in females and males following radiation exposure, we conducted bulk RNA sequencing (RNAseq) on the heart comparing control 0 Gy vs. 22 Gy females and males at day 10 and 35 in separate experiments. Differential gene expression analysis revealed that there were very few significantly different gene changes at day 35 in either sex (data not shown), indicating that DCM at day 35 is a cardiac functional endpoint with virtually no gene activity similar to our previous findings in a mouse model of myocarditis that progresses to DCM [30]. We initially conducted bulk RNAseq comparing males to control males at 22 Gy and later conducted the same analysis for females, so for this reason we cannot directly compare RNAseq between females and males.

#### Heatmaps, PCA and volcano plots at day 10

**Fig. 8:**
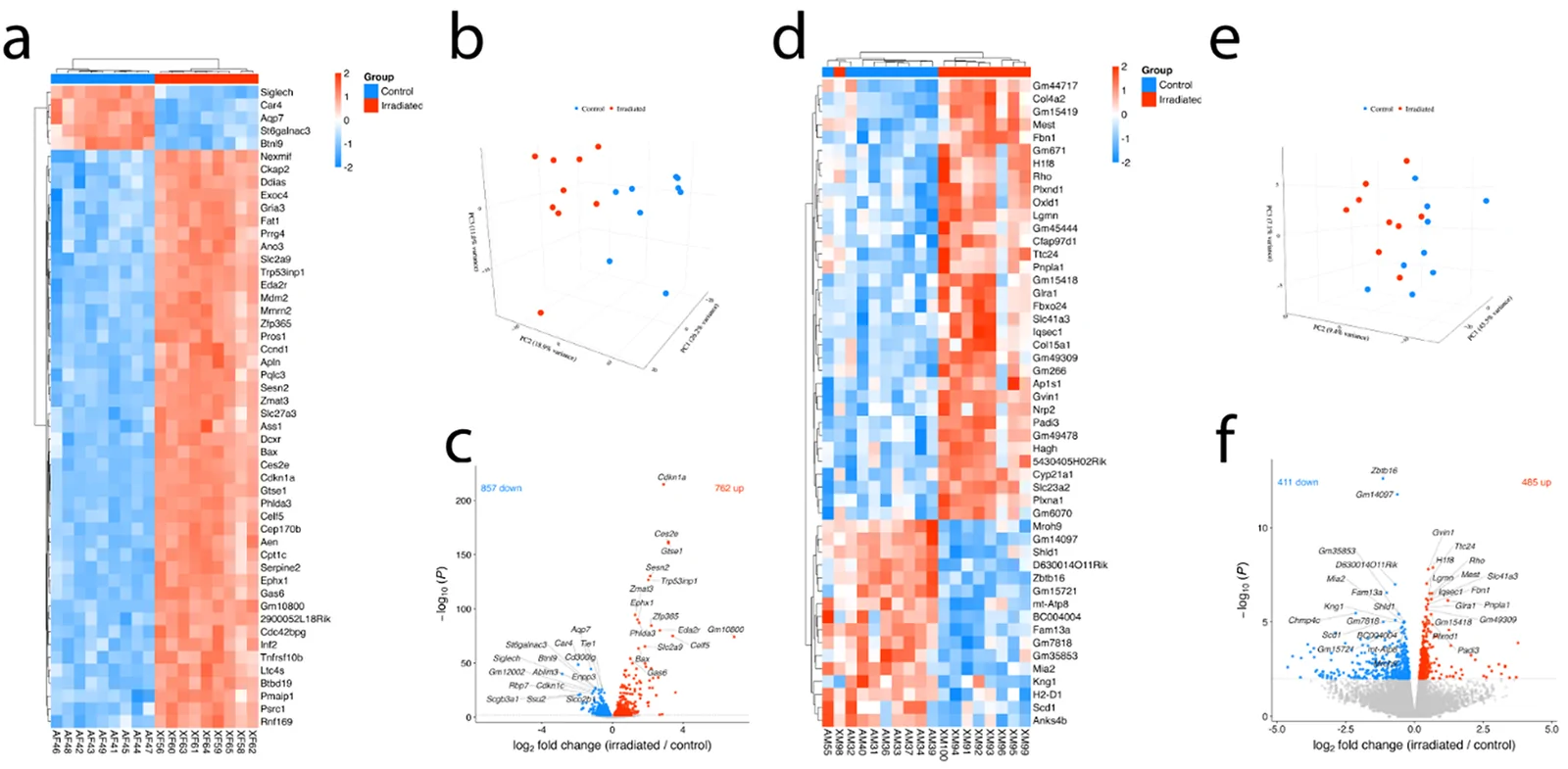
Characterization of gene changes in the heart of irradiated females and males vs. controls at day 10. Female and male BALB/c mice received 0 Gy (0Gy) or 22 Gy (22Gy) at day 0 and bulk RNA sequencing was performed on the heart at day 10 after irradiation in separate experiments (*n* = 8–9/group). Heatmap, principal component analysis (PCA) and volcano plots of females **a-c)** and males **d-f)** at day 10 after irradiation. A p value of 0.01 was used as the cutoff for genes shown in the volcano plots for **c)** females 857 down- and 762 up-regulated genes and **f)** males 411 down- and 486 up-regulated genes

Before analyzing the data, we performed quality control and preprocessing steps as described in the Methods (data not shown). Heatmaps of gene changes observed in the heart between 0 Gy control vs. 22 Gy females (Fig. 8a-c) and males (Fig. 8d-f) are shown for day 10 after irradiation. Principal component analysis (PCA) revealed distinct gene profiles between the two groups (Fig. 8b, e) and volcano plots indicate the top up- and down-regulated genes for each comparison (Fig. 8c, f).

#### WGCNA at day 10 in females

**Fig. 9:**
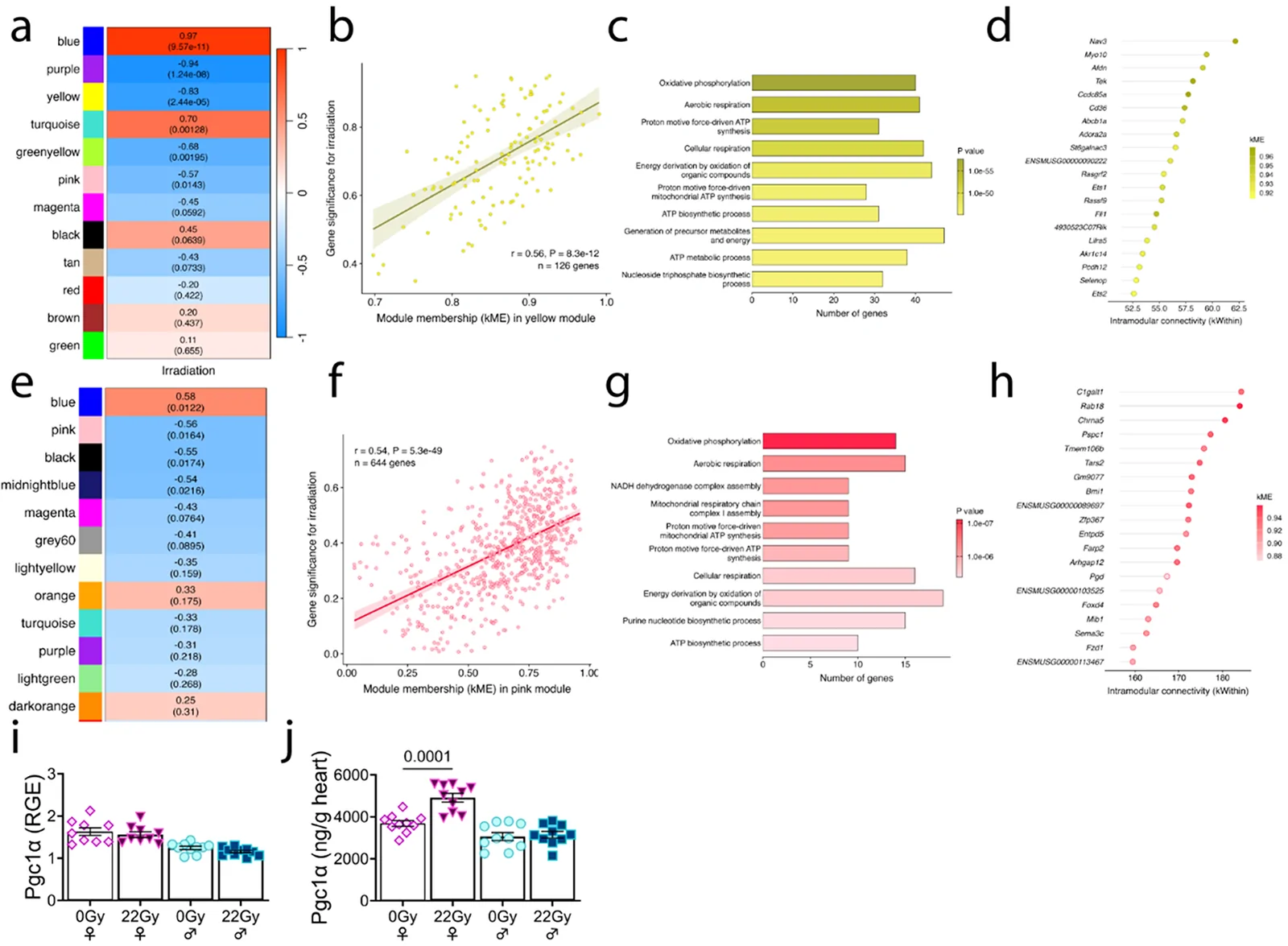
Weighted gene co-expression network analysis reveals radiation-induced mitochondrial dysfunction in females and males at day 10, but upregulation of Pgc1α, master regulator of mitochondrial function, in females. Female **(a-d)** and male **(e-h)** BALB/c mice received 0 Gy (0Gy) or 22 Gy (22Gy) at day 0 and bulk RNA sequencing was performed on the heart at day 10 after irradiation in separate experiments (*n* = 8–9/group). **a**,** e)** Module color assignments from high dimensional Weighted Gene Co-Expression Network Analysis comparing 0 to 22 Gy by sex revealed the top 12 up- (red) or down- (blue) regulated genes. Significantly down-regulated gene modules that related to mitochondrial function for **b)** females (yellow module, correlation = 0.56, *p* = 8.3 e-12, *n* = 126 genes) and **f)** males (pink module, correlation = 0.54, *p* = 5.3 e-49, *n* = 644 genes). Modules represent clusters of co-expressed genes associated with distinct transcriptional programs. Top 10 significant changed gene ontology (GO) pathways related to mitochondrial function in the **c)** yellow module in females and **g)** the pink module in males with oxidative phosphorylation and aerobic respiration being the top two GO pathways for yellow and pink. The top individual genes according to intramodular connectivity and kME are shown for **d)** yellow and **h)** pink modules. **i)** Relative gene expression (RGE) of *Pgc1α* levels normalized to the housekeeping gene *Hprt* in the heart at day 10 post radiation. **j)** ELISA of Pgc1α protein levels in the heart of homogenized hearts normalized to heart weight at day 10 after radiation. **i**,** j)** Data shows the mean ± SEM for 8–10 mice/group using 2-way ANOVA, p values obtained using Sidak’s multiple comparisons test

Analysis of RNAseq at day 10 using Weighted Gene Co-Expression Network Analysis (WGCNA) (Fig. 9) revealed that the most highly positively correlated gene module in females at day 10 related to radiation was the blue module (Fig. 9a). A strong positive correlation between blue module membership and gene significance was observed (correlation = 0.91, *p* < 1e-131) (data not shown). For the blue module in females, significant enrichment was observed in Gene Ontology (GO) pathways related to cellular structure and organization including “mitotic G1/S transition checkpoint signaling”, “positive regulation of cysteine-type endopeptidase activity involved in apoptotic process”, and “neural tube development”, for example (data not shown). These pathways suggest an increase in structural and signaling adaptation within the heart in females, likely as a response to radiation-induced stress or damage.

The most significantly negatively correlated gene module in females at day 10 was the yellow module (Fig. 9a), which also had a significant correlation (correlation = 0.56, *p* < 8.3e-12, *n* = 126 genes) (Fig. 9b). The top 10 GO pathways enriched in the yellow module all included mitochondrial metabolic pathways such as “oxidative phosphorylation”, “aerobic respiration,” and “proton-motive force driven ATP synthesis”, for example, suggesting that mitochondrial homeostasis was significantly impaired by radiation exposure at day 10 in females (Fig. 9c).

#### WGCNA at day 10 in males

Analysis of the RNAseq using WGCNA in males revealed that the most highly positively correlated gene module in males at day 10 related to radiation was the blue module (Fig. 9e). A strong positive correlation between blue module membership and gene significance was observed (correlation = 0.55, *p* < 1e-200) (data not shown). For the blue module in males, significant enrichment was observed in GO pathways related to cellular structure and organization including “establishment of organelle localization”, “cilium organization”, and “actin filament-based process regulation”, for example (data not shown). These pathways suggest an increase in structural and signaling adaptation within cells in males, likely as a response to radiation-induced stress or damage.

The most significantly negatively correlated gene module in males at day 10 was the pink module (Fig. 9e), which also had a significant correlation (correlation = 0.54, *p* < 5.3e-49, *n* = 644 genes) (Fig. 9f). The top 10 GO pathways enriched in the pink module all included mitochondrial metabolic pathways such as “energy derivation by oxidation of organic compounds”, “cellular respiration”, and “oxidative phosphorylation” (Fig. 9g), suggesting that mitochondrial homeostasis was significantly impaired at day 10 after radiation exposure in males. Thus, the gene profile at day 10 revealed upregulated cardiac repair mechanisms and dysregulation (i.e., downregulation) of mitochondrial pathways in both sexes following radiation exposure.

#### Cardiac vacuolization at day 10

We also observed increased cardiac vacuolization, which can be caused in part by mitochondrial damage, in both females and males at day 10 after irradiation. Representative photos are found in Supplemental Fig. 4.

### Females upregulate Pgc1α at day 10 after radiation suggesting possible mitochondrial repair

Pgc1α is a master regulator of mitochondrial function which improves mitochondrial function when it is elevated [39–41]. Although mitochondrial damage occurs following irradiation in both sexes as indicated by WGCNA, Pgc1α mRNA expression did not significantly differ between 0 and 22 Gy in either sex at day 10 (Fig. 9i). In contrast, cardiac Pgc1α protein levels measured by ELISA were elevated in females at day 10 after radiation (*p* = 0.0001) but not in males (Fig. 9j), suggesting that females may be better able to repair mitochondrial damage to the heart following radiation.

## Discussion

**Fig. 10:**
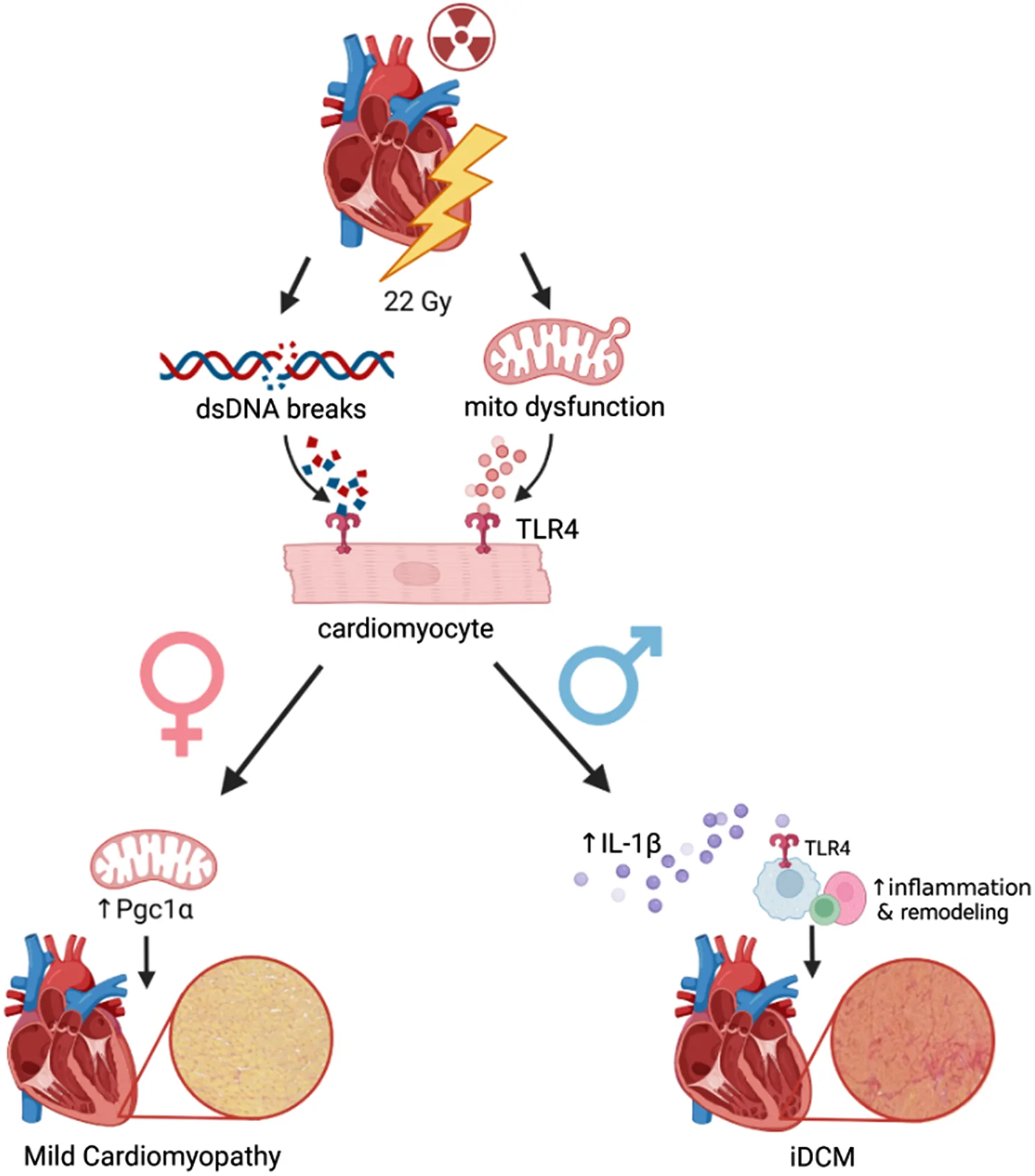
Graphical representation illustrating proposed mechanisms of sex differences in radiation-induced cardiomyopathy. Radiation directed to the heart leads to dsDNA breaks, mitochondrial dysfunction, vacuolation and vessel damage by day 10 after radiation in both males and females. Mitochondrial damage is known to upregulate TLR4 which leads to IL-1β production in the heart of males. Females may repair mitochondrial dysfunction by upregulating the master regulator of mitochondrial function, Pgc1α. Female hearts have less cardiac profibrotic cytokine IL-1β, less fibrosis and less chronic cardiac dysfunction than males. In contrast, males do not upregulate Pgc1α in response to mitochondrial damage. Instead, males have higher acute IL-1β that leads to fibrosis and iDCM after radiation

This study is the first, to our knowledge, to examine sex differences in RIHD in a translational mouse model. The main findings of this study were that radiation exposure to the heart led to cardiac dsDNA breaks, mitochondrial dysfunction, vacuolation and vessel hypertrophy and fibrosis by day 10 in both males and females (Fig. 10). Mitochondrial damage is known to release ATP and other mitochondrial components that activate TLR4 leading to IL-1β production [42, 43]. We found increased mitochondrial dysfunction and elevated IL-1β levels in males after radiation exposure. Females had elevated Pgc1α levels in the heart, which is an established indicator of mitochondrial homeostasis [44] suggesting a possible mechanism for improved mitochondrial repair in females. All these factors together may lead to better recovery from cardiac damage, and better cardiac function in females. In contrast, males did not upregulate Pgc1α in response to mitochondrial damage, suggesting worse mitochondrial repair following irradiation. In response to radiation damage males had elevated IL-1β and cardiac and perivascular fibrosis, resulting in iDCM by day 35 (Fig. 10). Future research is needed to establish the protective mechanism in females with RIHD.

Radiation is known to cause dsDNA breaks [45, 46], and so we examined this outcome as evidence that we had induced radiation damage in the heart. Surprisingly, we found that females had a higher number of dsDNA breaks based on γH2A.x expression at day 10 in the heart after 22 Gy irradiation. One possibility is that the ‘delivered’ dose differed by sex with females obtaining a greater area of damage because of their smaller heart size. However, this was largely accounted for by creating a dosimetric treatment plan according to sex. This plan focused radiation on the heart while minimizing exposure to the lungs for each sex. However, there was a slightly greater exposure to the heart and lungs of females based on the representative image shown in Fig. 1b and the dose-volume curves shown in Fig. 1c. Even though females had higher γH2A.x expression in the heart at day 10, males consistently developed worse cardiovascular outcomes than females in this study. Another possibility to account for these findings is that DNA repair mechanisms differ by sex. Studies suggest that women may be at significantly greater risk of suffering and dying from radiation-induced cancer than men exposed to the same dose of radiation [47]. A study of peripheral blood mononuclear cells (PBMCs) from healthy volunteers found no difference in γH2A.x expression by sex [48]. Similarly, a study by Fernandez et al. examined γH2A.x expression in PBMCs in patients with bladder cancer but did not observe sex differences [49]. In contrast, Mayer et al. found that DNA in human cells from males rejoin at a higher percentage of ds breaks than cells from females [50]. Importantly, even though females had higher γH2A.x expression in the heart at day 10, males consistently developed worse cardiovascular outcomes than females in this study.

Radiation exposure is also known to induce mitochondrial dysfunction [4]. Analysis of bulk RNAseq from whole hearts in this study found that both males and females developed mitochondrial dysfunction following 22 Gy irradiation. However, we observed elevated cardiac Pgc1α levels in irradiated females, but not in males. Pgc1α is a major regulator of mitochondrial homeostasis because it regulates the synthesis of mitochondrial proteins. Pgc1α was originally identified as a regulator of mitochondrial function in brown adipose tissue but was later also found to be expressed at high levels in cardiac tissue where it influences cardiovascular health and disease [51]. Pgc1α globally regulates mitochondrial pathways in response to stresses such as cold, fasting, infection and radiation [41, 52]. Sex differences in mitochondrial function in health and disease are also known [41, 53]. Naumenko et al. showed in 2022 in mice that cardiac-specific Pgc1α deficiency led to heart failure that occurred more quickly and was more severe in females than males, suggesting a greater protective effect of Pgc1α in females [53]. Estrogen (i.e., 17β-estradiol) has been shown to regulate Pgc1α expression in the heart [54]. We previously found that, following virus-induced mitochondrial damage, females with viral myocarditis exhibited higher cardiac Pgc1α expression than males, which was associated with reduced TLR4 and IL-1β expression, less fibrosis, and improved cardiac function [41]. Thus, the metabolic stress of radiation damage is a possible explanation for the elevated levels of Pgc1α that we observed in females after 22 Gy exposure that may have led to their improved cardiovascular outcomes. Future research is needed to establish the role of Pgc1α in better recovery in females following radiation exposure.

Sex differences in inflammation are well described [14, 55]. Females may be protected from many CVDs because the major immune factor that regulates inflammation, forkhead box P3 (FoxP3), is located on the X chromosome [55, 56]. Additionally, elevated 17β-estradiol levels in females further promote the expression of FoxP3- a marker of regulatory T cells (Tregs) [57]. Estrogen also drives a more regulatory phenotype in other immune cells such as macrophages, dendritic cells and T helper (Th)2/IL-4 responses [56, 58]. For example, cardiac inflammation in females with viral myocarditis have elevated anti-inflammatory alternatively activated M2 macrophages, Tim3^+^ CD4 T cells, Tregs and IL-4 [8]. Our findings in this study of an elevated CD11b^+^ and F4/80^+^ infiltrate in males following radiation exposure with elevated IL-1β levels, fibrosis and progression to iDCM is remarkably similar to the cardiac immune profile of males with viral myocarditis [8, 9], suggesting common pathogenic mechanisms following mitochondrial damage to the heart. Similar sex differences have been observed in patients with myocarditis [59]. Thus, our finding of iDCM in males with radiation exposure is consistent with other mouse models and clinical data that males are more likely to develop chronic cardiomyopathy and DCM compared to females. Future research is needed to determine whether RIHD in males is overlooked for more traditional explanations (i.e., diagnoses like atherosclerosis or idiopathic DCM).

There are several limitations to this study. Although we created a treatment protocol specifically for females vs. males, we did observe a slightly higher radiation exposure dose to the heart and lungs of female mice. Despite this difference, most of the cardiac outcomes we examined in this study were worse in males compared to females. In this study we provide echocardiography data comparing baseline to day 35 on the same mice because it is a better measure, from our experience, of changes in heart function because of the large variation in mouse heart size and orientation within the chest cavity in BALB/c mice. We provide a comparison of echo parameters in 0 Gy BALB/c mice aged 8–10 weeks (baseline) vs. 35 days (14 weeks) and find no significant difference in all echo parameters except for one (Supplemental Table 2), which suggests that the growth of the heart in one month in young mice has negligible effects on cardiac function. However, it is acknowledged that the heart size is likely to be larger at day 35 in both sexes compared to day 0 (baseline), which may have affected some echo calculations. Another limitation of this study is that we used only one dose of radiation. In contrast, patients typically receive multiple exposures of very small radiation doses over time. In the future, we would like to examine the effect of multiple small doses of radiation on the heart. However, the disadvantage in performing this approach in BALB/c mice is that mice must be anesthetized repeatedly and anesthesia affects cardiac function. In this study, we showed that RIHD can develop relatively quickly by day 35, similar to other models of DCM [32, 60] in mice that are susceptible to cardiomyopathy like BALB/c. Future studies should directly compare sex differences in cardiac gene expression during RIHD. Although we identified that females had increased levels of the master regulator of mitochondrial function Pgc1α, future studies are needed to determine the specific mechanisms that lead to less severe cardiomyopathy in females.

## Perspectives and significance

To our knowledge, this is the first study of sex differences in RIHD in mice. We found that females and males developed cardiomyopathy following radiation exposure, but sex differences were observed in the progression of disease. Mitochondrial dysfunction occurred in both sexes; however, females upregulated Pgc1α in the heart, a known regulator of cardiac mitochondrial homeostasis which may have improved disease progression in females. In contrast, males progressed to iDCM, which is known to occur more often in males. These findings highlight the clinical challenges in diagnosing RIHD, because it so closely resembles the outcome of other cardiovascular conditions. Translational mouse models that replicate clinical findings are important tools for identifying early biomarkers and potential therapies to improve outcomes in RIHD. Future studies should examine whether sex-specific biomarkers exist that could be leveraged to prevent progression to RIHD. These findings suggest that RIHD may be worse in male than female cancer survivors and future studies are needed that monitor disease progression following radiation treatment for cancer.

## Methods

### Animal care and ethics statement

Mice were used in accordance with recommended practices in the Guide for the Care and Use of Laboratory Animals of the National Institutes of Health (NIH), with approval obtained from the Institutional Animal Care and Use Committee (IACUC) at Mayo Clinic Florida for all procedures (IACUC# A00005356-20 and A00006478-22) as well as the Biosafety Committee.

### Mice

Adult female and male BALB/cJ (BALB/c) (strain #000651) 8-10-week-old mice were obtained from the Jackson Laboratory (Bar Harbor, ME) and maintained under pathogen-free conditions in the animal facility at Mayo Clinic Florida. We previously showed that we are powered to detect sex differences in cardiomyopathy with at least 7 mice/group [41, 61]. 7–10 mice per group were used for all experiments, unless otherwise indicated. Experiments were repeated to obtain all endpoints and ensure rigor. Tissues were collected at 24 h (innate) to examine immune cells and immune cell markers in the heart by qRT-PCR (qPCR), day 10 (acute) or day 35 post-irradiation (chronic). Numerous endpoints were examined at day 10 and 35 which are described in each figure legend.

### Radiation model

Male and female mice were randomly assigned to a group to receive: 0 Gy (control, anesthesia only, placed in X-ray machine without irradiation) or 22 Gy to the isocenter in a single cardiac dose. Whole heart irradiation was performed using the X-Rad SmART (Precision X-Ray, Madison, CT) small animal irradiator. Male and female mice were anesthetized using ketamine/midazolam (12.5 mg/mL; 0.6 mg/mL) and loosely wrapped in sterile pre-warmed fabric to prevent hypothermia inside of the irradiation chamber. Control (0 Gy) mice received ketamine/midazolam only and were placed in the device for the equivalent time as irradiated mice. Mice assigned to the radiation group (22 Gy) were placed in a feet-first prone position in relation to the gantry and two-dimensional computed tomography (CT) scans in the axial, sagittal and coronal views (40 kV, 0.8 mA) were generated. Computed tomography projections were loaded into the X-Rad SmART-ATP software (Precision X-Ray, Madison, CT) to create a sex-specific treatment plan for whole heart irradiation. A circular 5.0 mm (C5) collimator was used to deliver radiation to the heart and minimize damage to the surrounding tissues. The isocenter was placed in the center of the heart to deliver a radiation dose of 22 Gy x 1 fraction to the isocenter, with equally weighted parallel opposed beams. Because male and female mice differed in heart and thoracic anatomy, separate CT-based treatment plans were created to deliver the same prescribed 22 Gy dose to the isocenter while minimizing exposure to surrounding tissues. The treatment planning system generated plan-specific delivery parameters; consequently, dose rate and beam-on time differed between the female and male plans (Supplemental Table 1). These differences were outputs of the independently generated plans rather than different prescribed cardiac doses. An example setup and dose-volume histogram for the heart and lungs for male and female mice are shown in Fig. 1b, c.

### Histology

Murine cardiac tissue was dissected longitudinally along the sagittal plane at day 10 and day 35 after irradiation and fixed in 10% phosphate-buffered formalin for 48 h, shielded from light, and embedded in paraffin for histological analysis. 5 μm sections were stained with hematoxylin and eosin (H&E) to detect inflammation and vacuolation, and Masson’s trichrome to detect collagen. Cardiac inflammation and fibrosis were assessed as the percentage of the heart with inflammation or collagen deposition stained with H&E or Masson’s trichrome, respectively, normalized to the overall size of the heart section with the aid of a microscope eyepiece grid, as previously described [38, 60, 62].

### Immunohistochemistry

Embedded cardiac tissue sections were cut at 5 μm and stained with γH2A.x on day 10 post radiation (Cell Signaling Technology, Danvers, MA, USA 9718 S, 1:5000, rabbit). Envision+ anti-rabbit-labeled polymer (K4003) was used as the secondary antibody. IHC slides were scanned using an Aperio AT2 slide scanner (Leica, Wetzlar, Germany) and quantified as the number of strong positives (NSP) for γH2A.x divided by the total area (TA) of the section to calculate the percentage of positivity. The default “positive pixel” algorithm was modified by adjusting the hue width parameter so that the program’s selection of positive and negative pixel counted accurately reflected the stain. Positivity was determined using the default “Nsr” output (Nsr = number of strongly positive pixels/(number of weakly positive + number of positive + number of strongly positive pixels) where “strongly positive pixels” indicated intensity of the stain. The number of strong positives (NSP) from scanned cardiac tissues were assessed as a ratio of NSP compared to the total area (TA) of the section, as previously described [37].

### Echocardiography

Cardiac function was measured using transthoracic echo on day − 1 (baseline, 0 Gy) and day 35 (endpoint, 22 Gy) with the Vevo 3100 ultrasound machine equipped with a MX55OD 55 MHz transducer (VisualSonics Inc., Toronto, Canada), as previously described [31]. Anesthesia (12.5 mg/mL ketamine; 0.6 mg/mL midazolam) was administered by intraperitoneal injection and mice were placed in a supine position on a warming platform, with a rectal thermometer placed to monitor for hypothermia. Hair covering the thoracic region was removed using Nair and pre-warmed ultrasound gel (Parker Laboratories, Fairfield, NJ) was applied to the region. While under anesthesia, heart rate was monitored using the built-in echocardiography probes in the Vevo Lab Imaging Station and ranged from approximately 200 to 600 beats per minute (bpm) across animals (Supplemental Fig. 1). Two-dimensional (2D) parasternal long-axis (LAX) and short-axis (SAX) of the left-ventricle (LV) were acquired in B-mode. An M-mode image of the SAX was acquired by positioning the LV posterior wall at the level of the papillary muscles so both the free and septal wall were visible along with a portion of the right ventricle. The left ventricular (LV) end diastolic dimension (LVEDD), LV end systolic dimension (LVESD), LV anterior wall thickness at end diastole (LVAW;d) and end systole (LVAW;s), LV posterior wall thickness at end diastole (LVPW;d) and end systole (LVPW;s) were measured from a frozen M-mode tracing. Fractional shortening (FS %) is the percent change in LV cavity dimensions. Ejection fraction (EF %) represents stroke volume as a percentage of end diastolic LV volume. The heart rate is automatically determined from the M-mode image by positioning the first and second caliper point on two systolic phases. LV mass was determined automatically by the software or by using the following standard cube function formula: LV mass (mg) _ 1.055 [(IVST _ LVEDD_PWT)3_ (LVEDD)3] (1.055 is the specific gravity of the cardiac muscle). Relative wall thickness (RWT) indicates the overall thickness of the LV wall and is calculated as follows: (RWT) _ 2 _ PWTD/LVEDD, as previously described [32].

The probe was then placed to encompass the long-axis of the left ventricle and B-mode images and videos were captured for global longitudinal strain (GLS) analysis using Vevo Strain software, as previously described [27, 31, 41]. All strain measures were derived from the formula for cardiac strain which is defined by the difference in movement of a wall from its starting position at end-diastolic diameter to its end position at end-systolic diameter divided by the original position of the wall. This effectively represents a percent-change in wall position composed of individual component vectors. For 2D LAX this includes the longitudinal and radial movement vectors. Minimal adjustments were made to the speckle tracking and auto-LV artificial intelligence tracings when necessary and all analysis was performed while blinded to the treatment group.

### RNA isolation and qRT-PCR

At harvest, hearts were sectioned longitudinally along the sagittal plane to create two halves, snap frozen, and stored at -80℃. Half hearts were later homogenized and lysed using Tissuelyser (Qiagen, Hilden, Germany) with 7 mm stainless steel beads in RTL buffer with 0.5% DX buffer to reduce foam. RNA was isolated using Qiagen’s RNase Easy Fibrous Tissue Mini Kit (Qiagen, 74704). RNA concentration (Abs. 260) and quality (Abs. 260/280) were assed using a Nanodrop (Thermo-Scientific, Waltham, MA, 11095-080). RNA was converted to complementary DNA (cDNA) using iScript cDNA synthesis kit (Biorad, Hercules, CA, USA, #1708891), as previously described [41]. Two-step quantitative reverse transcriptase-mediated real-time PCR (qPCR) was used to measure abundance of individual mRNAs. Gene expression was measured using assay-on-demand primers and probe sets and the ABI7000 Taqman system (Applied Biosystems, Foster City, CA). Gene expression was assessed by comparative quantification, as previously described [38].

### RNA sequencing

Library preparation and bulk-tissue mRNA sequencing (RNAseq) for male and female control 0 Gy vs. 22 Gy irradiated hearts (*n* = 8–9/group) in separate experiments by sex were performed by the Mayo Clinic Genome Analysis Core. Analysis of the data was performed as previously reported. Libraries for this study used a standard poly A selection. RNA libraries were prepared using 200ng of total RNA according to the manufacturer’s instructions for the TruSeq Stranded mRNA Sample Prep Kit (Illumina, San Diego, CA). The concentration and size distribution of the completed libraries were determined using an Agilent Bioanalyzer DNA 1000 chip (Santa Clara, CA) and Qubit fluorometry (Invitrogen, Carlsbad, CA). Libraries were sequenced at 50 million fragment reads per sample following Illumina’s standard protocol using the Illumina cBot and HiSeq 3000/4000 PE Cluster Kit. The flow cells were sequenced as 100 × 2 paired end reads on an Illumina HiSeq 4000 using HiSeq 3000/4000 sequencing kit and HiSeq Control Software HD 3.4.0.38 collection software. Base-calling was performed using Illumina’s RTA version 2.7.7.

### Data pre-processing

Aligned cram files were generated by the Mayo Clinic Genome Analysis Core from the RNAseq data, which were then processed to quantify gene expression levels. RNAseq read count processing was performed on these cram files using the featureCounts tool from Subread v2.0.3 with the Gencode vM30 annotation GTF. Lowly expressed genes (mRNA count < 10) were filtered out and the remaining counts normalized downstream using DESeq2 v1.44.0. The resulting count matrix was subsequently utilized for downstream differential expression analysis and functional enrichment analysis using appropriate statistical methods.

### Differential gene expression analysis

For differential expression analysis, we utilized edgeR version 4.2.2 on two separate RNAseq datasets collected at day 10 or 35. Each dataset consisted of gene expression counts across samples from two groups: control (0 Gy) and treatment (22 Gy). Raw count data and corresponding metadata for each day were imported from CSV files. The raw count data contained expression counts for each gene across samples, while the metadata included sample information such as group and time point. Separate DGEList objects were created in edgeR for each dataset (day 10 and 35), encapsulating the count data and group information.

To ensure reliable differential expression analysis, lowly expressed genes were filtered out in each dataset using the filterByExpr function, retaining only genes with adequate counts across samples. After filtering, the DGEList objects were updated, and library sizes were recalculated to exclude lowly expressed genes. Normalization factors were then calculated using the calcNormFactors function to adjust for differences in library sizes and composition across samples, ensuring fair comparisons of expression levels between samples within each dataset. Biological variability across samples was accounted for by estimating dispersion for each gene in both datasets using the estimateDisp function. Dispersion estimation is essential for accurately modeling count data variability, particularly for experiments with biological replicates. Each DGEList object was updated with these dispersion estimates to improve the robustness of downstream analyses. Differential expression testing was then performed separately for each dataset using the exactTest function, which conducts a statistical test for differences in gene expression between the control (0 Gy) and treatment (22 Gy) groups. This approach is based on the negative binomial distribution and incorporates the dispersion estimates calculated for each dataset. Gene IDs in the original count data were provided as murine Ensembl gene IDs (ENSMUSG). These IDs were converted to gene symbols using the biomaRt package version 2.60.1 in conjunction with the org.Mm.eg.db database version 3.19.1, allowing for easier interpretation and compatibility with annotation databases. The results of the differential expression analysis were extracted using the topTags function, providing a ranked list of genes based on statistical significance for each time point. We also used the decideTests function to generate a summary of significant genes, indicating the number of upregulated and downregulated genes for each dataset. The full differential expression results, including fold changes, p-values, and adjusted p-values (FDR), were saved as separate CSV files for each time point (day 10 and 35). These files contain information on all tested genes, enabling further interpretation and validation of differentially expressed genes. This edgeR workflow allowed us to identify genes with significant expression changes associated with the treatment groups over time, providing insights into the molecular response at each time point.

### Co-expression network analysis

To identify gene co-expression modules and their association with experimental traits, we used the Weighted Gene Co-expression Network Analysis (WGCNA) R package v1.73. We filtered genes to include only those with significant variability across samples. The data were then normalized, and soft-thresholding powers (β) of 3 for day 10 and 4 for day 35 were selected to achieve approximate scale-free topology fits of R² > 0.85 for day 10 and 0.8 for day 35, as determined by the criterion of approximate scale-free topology. Using these selected β values, we transformed the similarity matrices into adjacency matrices, capturing gene connectivity based on co-expression. These were further converted into Topological Overlap Matrices (TOMs) to enhance robustness by incorporating shared connections between genes. Gene modules were identified by hierarchical clustering on the TOM-based dissimilarity, followed by dynamic tree cutting to delineate distinct modules, with each module assigned a unique color for easy visualization. To explore the relationship between modules and the trait of interest, we computed module eigengenes (the first principal component of each module) to summarize the expression profile of each module. We then correlated these module eigengenes with the external trait (“Radiation”), identifying modules significantly associated with the trait. These relationships were visualized in a heatmap, where significant modules exhibited strong positive or negative correlations. For modules with significant correlations, we created scatter plots of module membership (correlation between each gene and the module eigengene) against gene significance (correlation between each gene and the trait) to assess the strength of association between module relevance and trait significance. To interpret the biological relevance of each gene module, we conducted pathway enrichment analysis using the Gene Ontology (GO) database. Genes from each module were extracted and converted to gene symbols or Entrez IDs, as required for pathway analysis tools, and only genes from modules significantly correlated with the trait were included in the enrichment analysis. The clusterProfiler R package v4.12.6 was employed to test for the overrepresentation of GO terms (Biological Process) in each module. This analysis used a hypergeometric test, excluding GO terms with fewer than 5 or more than 500 genes to avoid non-specific results. P-values from the enrichment analysis were adjusted for multiple testing using the Benjamini-Hochberg procedure, with a false discovery rate (FDR) threshold of 0.05 for significance. For each significant module, the top 10 enriched pathways were visualized in bar plots using ggplot2 v3.5.1, where bar lengths represented the number of genes associated with each pathway and colors indicated the level of statistical significance (p-value). Pathways with significant enrichment (FDR < 0.05) were interpreted to provide insights into the biological functions associated with each module. This workflow enabled the identification of biologically relevant gene modules and the elucidation of their functional roles through pathway enrichment, offering insights into the underlying molecular processes associated with the trait of interest.

### Vessel measurements

Vessel wall thickness (VWT) was determined from H&E-stained slides by measuring the width of 3 vessels/sample using ImageScope at high magnification at the same 4 locations for each vessel. Vessel area (%) was obtained by taking the area of the outer lumen minus the area of the inner lumen x100 using ImageScope of 4 vessels/sample. Perivascular fibrosis was calculated from Masson trichrome stained slides by tracing the vessel (inside) and tracing the area of perivascular fibrosis (outside) and then calculating the area.

### ELISA

Frozen hearts were rapidly thawed and weighed to obtain tissue wet weight before homogenizing using a polytron homogenizer in minimum essential media (MEM) with 2% fetal bovine serum (FBS). Homogenized tissue was centrifuged at 3,000 rpm at 6 °C for 20 min and the supernatant was collected for analysis. Protein expression was quantified using the mouse peroxisome proliferator-activated receptor gamma coactivator 1-alpha (PPARGC1A) (note same as Pgc1α) ELISA Kit (MyBioSource, cat#MBS707053, San Diego, CA), and mouse IL-1 beta/ IL-1F2 Quantikine ELISA Kit (cat#MLB00C), mouse TNFα Quantikine ELISA Kit (cat#MTA00B-1), and Human/ Mouse/ Rat/ Porcine/ Canine TGF-β1 Quantikine ELISA kit (cat# DB100C) (R&D Systems, Minneapolis, MN). Absorbance was used to calculate concentration relative to a standard curve and normalized to tissue wet weight, as previously [37, 38, 62, 63]. The lowest detection limit for the Pgc1α kit was 6.0 pg/mL, IL-1β was 4.8 pg/mL, TNFα was 1.9 pg/mL, and TGFβ1 was 2.4 pg/mL.

### Statistical analysis

Normally distributed data comparing two groups were analyzed using a 2-tailed Student’s *t* test. The Mann-Whitney rank sum test was used to evaluate nonparametric data. Data are expressed as mean ± SEM. When comparing more than 2 groups multiple comparison analysis was performed by ANOVA (and nonparametric equivalent for nonparametric data) with each group compared to the corresponding control group; 2-way ANOVA examined the effect of radiation and sex. We previously showed that we are powered to detect sex differences in cardiac diseases with at least 7 mice/group [41, 61]. 7–10 mice per group were used for all experiments, unless otherwise indicated. Data are expressed as mean ± SEM. A value of *p* < 0.05 was considered significant.

## Supplementary Information

Below is the link to the electronic supplementary material.

Supplementary Material 1

## Data Availability

Data used for RNA sequencing and subsequent WGCNA are available upon request. Other requests for data used in this manuscript can be addressed to the corresponding author and are available upon reasonable request.

## Abbreviations

ANOVA: Analysis of variance
BW: Body weight
CT: Computed tomography
CVDs: Cardiovascular diseases
DCM: Dilated cardiomyopathy
ds: Double-stranded
DVH: Dose-volume histogram
Echo: Echocardiography
ELISA: Enzyme linked immunosorbent assay
FoxP3: Forkhead box P3
FS: Fractional shortening
GLS: Global longitudinal strain
GO: Gene Ontology
Gy: Gray
H&E: Hematoxylin and eosin
WGCNA: Weighted Gene Co-Expression Network Analysis
HF: Heart failure
HFpEF: Heart failure with preserved ejection fraction
HFrEF: Heart failure with reduced ejection fraction
IACUC: Institutional Animal Care and Use Committee
iDCM: Inflammatory dilated cardiomyopathy
IHC: Immunohistochemistry
IL: Interleukin
LV: Left ventricular
LVEDD: Left ventricular end diastolic diameter
LVEDV: Left ventricular end diastolic volume
LVEF: Left ventricular ejection fraction
LVESD: Left ventricular end systolic diameter
LVESV: Left ventricular end systolic volume
NIH: National Institutes of Health
NSP: Number of strong positives
PBMCs: Peripheral blood mononuclear cells
qPCR: quantitative RT-PCR
RIHD: Radiation-induced heart disease
RNAseq: RNA sequencing
Th: T helper
TL: Tibia length
TLR4: Toll-like receptor 4
Tregs: Regulatory T cells
VWT: Vessel wall thickness
