# Supplementary material for: Sex differences in a mouse model of radiation-induced cardiomyopathy
Source: Biol Sex Differ. 2026 Oct 8;17:166. doi: 10.1186/s13293-026-00998-3 (PMC13660165; doi:10.1186/s13293-026-00998-3)
Supplement: Supplementary file 1 [file 13293_2026_998_MOESM1_ESM.docx]

***Supplemental Materials***

**Sex differences in a mouse model of radiation-induced cardiomyopathy**

Emily R. Whelan^1-3*^, Evan E. Perona^1*^, Jessica J. Fliess^1^, S. Christian Kocsis^1^, Gabriel J. Weigel^1^, Elizabeth J. McCabe^1^, Charwan Hamilton^1^, Damian N. Di Florio^1-3^, Varsini Balamurugan^1^, Danielle J. Beetler^1-3^, Logan P. Macomb^1,4^, Max W. Strandes^1^, Frances C. Wilson^1^, Emma F. Murphy^1^, Ashley A. Darakjian^1^, Molly M. Watkins^1-3^, Alayna M. Puls^1^, Cameron J. Hartmoyer^1^, Natalie Bonvie-Hill^1^, Sami Khatib^1^, Chris J. McLeod^1^, Jordan C. Ray^1^, Michael D. Story^4^, Steven M. Herchko^4^, Bradford S. Hoppe^4^^, DeLisa Fairweather^1,2,5^^#

^1^Department of Cardiovascular Medicine, Mayo Clinic, Jacksonville, Florida, USA

^2^Center for Clinical and Translational Science, Mayo Clinic, Rochester, Minnesota, USA

^3^Mayo Clinic Graduate School of Biomedical Sciences, Mayo Clinic, Jacksonville, Florida, USA

^4^Department of Radiation Oncology, Mayo Clinic Jacksonville, FL, USA

^5^Department of Immunology, Mayo Clinic, Jacksonville, Florida, USA
*Co-first authors
^Co-senior authors

**#Correspondence:** DeLisa Fairweather, PhD, FAHA, FHFSA
Phone: 904-953-6740
Fax: 904-953-2439
Email: [Fairweather.DeLisa@mayo.edu](mailto:Fairweather.DeLisa@mayo.edu)

**Supplement Tables**

Supplemental Table 1. Dosimetric treatment plan for male and female BALB/c mice.

|  | Gy/min | Total Beam Time (min) | Beam Weight | Peak Voltage (kV) | Tube Current (mA) | Filtration | Delivery Angles |
| --- | --- | --- | --- | --- | --- | --- | --- |
| *Females* | 2.606 | 8.442 | 1:1 | 225 | 13 | 0.15mm Cu | 90° & 270° |
| *Males* | 2.698 | 8.152 | 1:1 | 225 | 13 | 0.15mm Cu | 90° & 270° |

Supplemental Table 2. M-mode echocardiographic parameters in female and male 0 Gy controls comparing baseline (day 0) and endpoint (day 35) values.

| Parameter | F-0Gy-B* | F-0Gy-E | *p* | M-0Gy-B | M-0Gy-E | *p* |
| --- | --- | --- | --- | --- | --- | --- |
| *Heart Rate* (bpm) | 379.19(34.03) | 296.06(30.74) | 0.098 | 374.08(42.99) | 363.21(32.56) | 0.831 |
| *LVEF* (%) | 68.15(2.91) | 64.10(2.83) | 0.131 | 66.48(3.79) | 64.52(3.55) | 0.731 |
| *FS* (%) | 37.30(2.06) | 34.78(2.22) | 0.220 | 36.64(3.05) | 35.27(2.73) | 0.761 |
| *LVESD* (mm) | 1.96(0.12) | 2.20(0.13) | 0.225 | 2.21(0.20) | 2.47(0.23) | 0.353 |
| *LVEDD* (mm) | 3.11(0.11) | 3.36(0.13) | 0.306 | 3.43(0.18) | 3.77(0.26) | 0.244 |
| *SV* (µL) | 25.90(1.69) | 29.76(2.68) | 0.422 | 31.76(2.50) | 40.11(5.66) | 0.177 |
| *CO* (µL/min) | 9.69(0.94) | 8.57(0.88) | 0.447 | 11.67(1.60) | 13.69(1.77) | 0.356 |
| *LVESV* (µL) | 12.92(2.13) | 17.40(2.63) | 0.247 | 18.33(3.85) | 24.38(4.71) | 0.290 |
| *LVEDV* (µL) | 38.82(3.30) | 47.17(4.79) | 0.322 | 50.09(5.88) | 64.49(9.62) | 0.160 |
| *LV Mass* (mg) | 92.80(10.90) | 107.97(12.45) | 0.531 | 78.27(9.34) | 101.90(11.60) | 0.113 |
| *LV Mass Corr.* (mg) | 74.24(8.72) | 86.38(9.96) | 0.531 | 62.61(7.47) | 81.52(9.28) | 0.113 |
| *LVAW;s* (mm) | 1.26(0.07) | 1.19(0.07) | 0.670 | 1.15(0.09) | 1.16(0.07) | 0.908 |
| *LVAW;d* (mm) | 0.90(0.11) | 0.75(0.06) | 0.436 | 0.71(0.06) | 0.73(0.05) | 0.823 |
| *LVPW;s* (mm) | 1.21(0.08) | 1.34(0.16) | 0.573 | 0.94(0.09) | 1.28(0.07) | **0.015** |
| *LVPW;d* (mm) | 0.88(0.07) | 1.07(0.16) | 0.422 | 0.69(0.08) | 0.81(0.06) | 0.169 |

*B, baseline at day 0; E, endpoint at day 35; F-0Gy, females 0 Gy; M-0Gy, males 0 Gy; *p*, p value.

**Supplemental Figures**

**Supplemental Figure 1. Other echocardiographic parameters.** The cardiac function of female and male BALB/c mice was assessed at day 0 (baseline, 0Gy) and 35 after receiving 22 Gy (22Gy) for the following parameters in separate experiments for females and males: a) heart rate, b) left ventricular (LV) mass, c) LV mass corrected (Corr.), d) LV anterior wall thickness in systole (LVAW;s), e) LV anterior wall thickness in diastole (LVAW;d), f) LV posterior wall thickness in systole (LVPW;s), and g) LV posterior wall thickness in diastole (LVPW;d). Data show the mean ±SEM for *n* = 8-13 mice/group, p values obtained using Wilcoxon matched-pairs signed rank test. There were no significant differences between groups.

**Supplemental Figure 2. TLR4 time-course in the heart following irradiation.** Female and male BALB/c mice received 0 Gy (0Gy) or 22 Gy (22Gy) at day 0 and *Tlr4* levels in the heart were examined at **a)** 24 hours (24H), **b)** day 10 (d10) and **c)** day 35 (d35) after irradiation using qPCR relative to the gene expression (RGE) of the housekeeping gene *Hprt*.

**Supplemental Figure 3. Levels of other profibrotic cytokines in the heart at day 10 after irradiation.** ELISA of **a)** TNFα, **b)** TGFβ1 levels in the heart at day 10 after 22 Gy radiation vs. 0 Gy normalized to heart weight. Data shows the mean ±SEM for 8-10 mice/group using 2-way ANOVA, p values obtained using Sidak’s multiple comparison’s test.

**Supplemental Figure 4. Radiation increased cardiac vacuolization at day 10.** Male and female BALB/c mice received 0 or 22 Gy radiation on day 0 and hearts were examined at day 10 post radiation. Representative histology photos in males using H&E stain compare **a)** 0 Gy vs. **b)** 22 Gy hearts which displayed greater vacuolization (white spaces, arrows) after radiation (Bar = 60µm).
